# Supplementary material for: Exploring the path to corruption–An informed grounded theory study on the decision-making process underlying corruption
Source: PLoS One. 2023 Sep 21;18(9):e0291819. doi: 10.1371/journal.pone.0291819 (PMC10513331; doi:10.1371/journal.pone.0291819)
Supplement: S1 Appendix — (DOCX) [file pone.0291819.s001.docx]

**Appendix**

Interview Guide

*Examples of Pre-Prepared Questions*

1. What behavior of yours was regarded by the judges as violating the law?
2. What was your goal when you decided act like that?
3. Before you decided to act like that, did you look for any kind of information to base your decision on?
4. What kind of information did you search for?
5. What was the most important factor that you considered when you finally made the decision to act like that?

*Examples of Questions Added Later Based on Prior Interviews*

1. What was your role in that situation?
2. So, what was your fault?
3. What was the benefit for you of carrying out that behavior?
4. Why did you choose that behavior and not another?
