# Supplementary material for: Exploring the path to corruption–An informed grounded theory study on the decision-making process underlying corruption
Source: PLoS One. 2023 Sep 21;18(9):e0291819. doi: 10.1371/journal.pone.0291819 (PMC10513331; doi:10.1371/journal.pone.0291819)
Supplement: S1 File — (PDF) [file pone.0291819.s002.pdf]

18/08/2023

Coding Dictionary: Exploring the path to corruption – An informed grounded theory study on the decision-making process underlying corruption

Table of contents

1. Documents 6
2. Goal 8
  2.1. Personal gain (money) 8
    2.1.1. Consumptive needs 8
    2.1.2. Money for having fun 8
    2.1.3. Pay previous debt 8
    2.1.4. Personal assets 9
    2.1.5. Money for my research 9
    2.1.6. Expenses for my family 9
    2.1.7. Getting money 10
    2.1.8. Getting the profit 13
  2.2. Personal gain (self-esteem) 13
    2.2.1. Showing ability 13
    2.2.2. Pride 13
    2.2.3. Being appreciated 14
    2.2.4. Being popular and remembered 15
  2.3. Personal gain (job) 15
    2.3.1. Doing the jobs description 15
    2.3.2. Job security 16
    2.3.3. I wanted my company to be a big company 16
    2.3.4. Career development 16
    2.3.5. Getting the jobs/projects 17
    2.3.6. For my company 17
  2.4. Society/Community 18
    2.4.1. Helping friend 18
    2.4.2. Helping the farmers 19
    2.4.3. My village get funding again 19
    2.4.4. For the public facility 20
    2.4.5. For the people 21
    2.4.6. My village 21
  2.5. Organization 22
    2.5.1. Supporting regional government project 23
    2.5.2. Organizational profit 23
    2.5.3. Organization reputation 23

2.5.4. To build the market without spending any money 24
2.5.5. Making money kept on growing 24
2.5.6. Employee's sake 25
2.5.7. Promoting the organization 25
2.5.8. To get the project (organization performance) 25
2.5.9. Organizational profit/income 26
3. Information content 27
3.1. Open focus 27
3.1.1. Coordinating about do or don't do it 27
3.1.2. Solution search/way 28
3.1.3. Legal status 29
3.2. Corruption focus 30
3.2.1. Comparing (what others do) 30
3.2.2. Legal consequence 33
3.2.3. The way (how to) 33
3.2.4. Safety 36
4. Information source 38
4.1. Impersonal sources 38
4.1.1. Within organization 38
4.1.2. Analyst report 39
4.1.3. Media 39
4.1.4. Legal authority 40
4.2. Interpersonal sources 42
4.2.1. Self-knowledge/experiences 42
4.3. Intrapersonal sources 43
4.3.1. Colleague in the other organization 43
4.3.2. Informal relations 46
5. Consideration/Evaluation 47
5.1. Push reasons 47
5.1.1. Rely on the others 47
5.1.2. That's the way it should be 53
5.1.3. Doing the instruction 56
5.1.4. Because I already went into the wrong way 59
5.2. Pull reasons 59
5.2.1. It was not corruption 59
5.2.2. Secure/safe 67

5.2.3. Common practice/Others also did ... 77
5.2.4. Easy/simple way ... 80
5.2.5. Change ... 83
6. Behavior ... 84
6.1. Conspiracy victim ... 84
6.1.1. Being appointed as project committee ... 84
6.1.2. Not involved in the project committee ... 84
6.1.3. Selling the land to the state company ... 85
6.1.4. Giving the loan ... 85
6.1.5. Borrowing money to the state company ... 85
6.1.6. Assisting farmer community for productivity ... 85
6.1.7. Buying the land for the sugar factory ... 85
6.1.8. Being considered as the state company staff ... 86
6.2. Wrong policy/administration ... 86
6.2.1. Signing contract for exploring natural resource for private co ... 86
6.2.2. Building village market in the disputed land ... 86
6.2.3. Giving the money for a project without a proper receipt ... 86
6.2.4. Spending money that was not included in the budget ... 86
6.2.5. Didn't realize the project grant ... 87
6.2.6. Land acquisition without appraisal process ... 87
6.2.7. Investing money to the business outside organization's vision ... 88
6.2.8. Borrowing money to the bank with wrong procedure ... 88
6.2.9. Giving the credit without proper management ... 88
6.2.10. Signing the receipt for the old project ... 89
6.3. Favouritism ... 89
6.3.1. Harming the organization and giving the benefit to other party ... 89
6.3.2. Giving the loan from organizational budget to the football club ... 89
6.3.3. Giving the loan to the ineligible people ... 89
6.3.4. Giving the loan from organizational budget to small enterprise ... 90
6.4. Assisting/involved ... 90
6.4.1. Assisting in money movement process ... 91
6.4.2. Making the false document ... 92
6.4.3. Helping in land acquisition process ... 93
6.4.4. Lending the company for the corrupt project ... 93
6.4.5. Managing the event ... 94
6.4.6. Finding institution as partner in corruption process ... 94

6.4.7. Falsifying financial report 95
6.4.8. Signing the document 95
6.4.9. Getting money from the failed credit 96
6.4.10. Helping in the finding the new creditors 96
6.5. Bribe 97
6.5.1. Giving bribe 97
6.6. Manipulation of information 98
6.6.1. Mark-up 98
6.6.2. Making the false document 98
6.6.3. Falsifying creditor identity 98
6.6.4. Manipulating the data to get the loan 98
6.6.5. Building the road was not according to the grant agreement 99
6.6.6. Falsifying the financial report 99
6.6.7. Executed the event that was not according to the budget plan 100
6.7. Embezzlement 101
6.7.1. Borrowing money from the project budget 101
6.7.2. Taking the money 101
6.7.3. Getting/obtaining money from the project 102
6.7.4. Taking money from the government budget 103
6.7.5. Mark-up 104
6.7.6. Getting money from the failed credit 105

1. Documents

Participant ID	Document
1	REC01
2	REC02_translated_checked
3	REC03
4	REC04
5	REC05
6	REC06_translated_checked
7	REC07
8	REC08
9	REC09
10	REC10_translated_checked
11	REC11 checked_translated 171104
12	REC12
13	REC13
14	REC14
15	REC15
16	REC16
17	REC17
18	REC18
19	REC19
20	REC20
21	REC21
22	REC22
23	REC23
24	REC24
25	REC25
26	REC26
27	REC27
28	REC28
29	REC29A
29	REC29B

30	REC30A
30	REC30B
31	REC31
32	REC32_translated_checked
33	REC33 checked_translated 171114
34	REC34
35	REC35
36	REC36
37	REC37
38	REC38

Note. The numbers behind the document names are participant IDs.

2. Goal

2.1. Personal gain (money)

2.1.1. Consumptive needs

1.

"Iya Konsumtif cendrung, iya kan? Iya Konsumtif. Bukan ekonomi dalam artian Basic Need. Ini bukan Basic Need lagi. Kalau ini sudah kaya kok. Kita kan ga berfikir “apa yang mau dimakan besok?”, “saya nanti mau makan apa?” kan begitu? "

Code: Consumptive needs
REC01

2.

"Kebutuhan tadi itu bukan basic need lagi tapi kebutuhan yang konsumtif."

Code: Consumptive needs
REC01

3.

" Iya kebutuhan. Tapi bukan kebutuhan mendasar iya. Kebutuhan uang segini, bisa begini, bisa beli mobil, bisa mobil baru. "

Code: Consumptive needs
REC01

4.

Code: Consumptive needs
REC34

2.1.2. Money for having fun

1.

"Waduh, kalau kebutuhanya, iya namanya waktu itu saya masih muda, iya kan. Masih pengaruh dunia luar, yoo masih seneng-seneng di luar atau apa, kan gitu aa, ndak terasa. Memang dalam waktu sekian jam itu sudah habis."

Code: Money for having fun
REC04

2.

"Ayo mas jalan-jalan? Ayo mas nongkrong di sana?” ndak ada program yang menyenangkan yang sekiranya untuk bangun, untuk dirinya sendiri itu loo, untuk bangun rumah ku sekiranya ndak bagus, ndak ada. Hilangnya uang segitu itu hanya untuk gengsi-gengsian aja. Ini loo pengaruhnya di situ. Jadi ga bisa pegang yang sudah dipercayakan."

Code: Money for having fun
REC04

2.1.3. Pay previous debt

1.

". Separuhnya untuk nasabah sendiri dan separuhnya saya pakek untuk menutup setoran-setoran yang jadi tanggung jawab saya. Jadi gali lobang , tutup lobang. Jadi pinjem nama gitu"

Code: Pay previous debt
REC25

2.

"Sebenarnya kalo tujuan menutup setoran yang menjadi tanggung jawab saya. Setoran angsuran kan tanggung jawab bendahara gaji. Jadi, gimana cara saya mencari uang untuk setoran itu tadi biar tidak ketahuan kantor. Misalnya, ketahuan kantor, kok setorannya tidak ada pembayaran di koperasi , otomatis saya ketahuan. Saya menutupi itu semua selam 2 tahun dari pimpinan, temen kantor kecuali teman saya yang 2 ini. "

Code: Pay previous debt
REC25

3.

"Murni untuk setoran koperasi yang menjadi tanggung jawab saya. "

Code: Pay previous debt
REC25

4.

"Nah, kondisi ini membuat hutang saya semakin menumpuk, terus akhirnya saya meminjam uang di bank. Waktu itu saya dinas di suatu instansi, disebutkan ya?
P He'em.
R Di instansi Kecamatan x. Tapi gaji saya sendiri sudah habis di situ, jadi saya minta tolong teman di kecamatan lain untuk meminjamkan uang di bank. Atas nama saya seakan-akan saya adalah pegawai di kecamatan lain tersebut. Saya berdinas di kecamatan x minta teman saya di kecamatan x."

Code: Pay previous debt
REC27

2.1.4. Personal assets

1.

"Memang benar, saya tidak ingin menutupi bahwa sebagian dari pinjaman itu juga saya gunakan untuk beli tanah atau rumah. "

Code: Personal assets
REC27

2.

"Terus kemudian, kalo Allah mengijinkan waktu itu tak ada korupsi, mungkin kita dapet CV"

Code: Personal assets
REC38

2.1.5. Money for my research

1.

"Yaa, karena untuk mencari paten tak cukup, pengembangan tak ada dana, pengembangan cari sendiri."

Code: Money for my research
REC19

2.1.6. Expenses for my family

1.

"That was how I ended up like that, and eventually it came true [got in to prison]. If I didn't do it that way, then I went into jail, what would my wife and child expenses, sir"

Code: Expenses for my family
REC10_translated_checked

2.

"Nothing was confiscated, thank God, we can use it for my wife and children's life.)"

Code: Expenses for my family
REC10_translated_checked

3.

"Thank God they believe, because in fact is that I took everything for my child and wife. Not for my own sake.)"

Code: Expenses for my family
REC10_translated_checked

4.

" Oh well. After all, I have family to look for, to feed. I thought about their life"

Code: Expenses for my family
REC10_translated_checked

5.

"I only thought about my family. Whoa! I got arrested, that's life. Anything could happen when I'm in here. What if suddenly something happen? If someone gets sick, but I'm here, what should my wife do? I only thought of that. Sometimes I was afraid, I mean, my wife and I were young, and as a man I'm afraid to let her down. "Oh man! My wife need this and that, it's gonna be hard for me. That was my only reason. I just want to be responsible [as a husband]. "

Code: Expenses for my family
REC10_translated_checked

6.

Code: Expenses for my family
REC28

2.1.7. Getting money

1.

"No I don't. I did get some from people who sold it. When I picked up and delivered [some documents], then I received some money. They gave me 200-300 [thousand]. Let's say someone asked me, "I'd like to sell mine" then I put him in the list. They came to him and he gave me some commission. "Okay, if it's a deal, then I'll give you some commission" "Okay". Then the village secretary's representative came to his house, after they made a deal, he gave me the commission. I didn't participate in the negotiation. [For the negotiation] the other officer would come"

Code: Getting money
REC02_translated_checked

2.

"Seharusnya saya ndak boleh menerima pemberian itu, ndak boleh. Maksimal pemberian yang diberikan kepada itu harusnya paling banyak 2 juta, pekerjaan satu tahun itu. Tapi pada saat itu tidak saya jelaskan saya diberi siapa. Total semua itu saya diberi masyarakat juga, masyarakat itu banyak, puluhan orang, nah itu saya dikasih begitu juga dari kampus x saya kumpulkan jadi satu ada sekitar 76 juta itu. Lah sekarang, pada saat itu masyarakatnya sekitar 74 orang. Lah kalau per orang ngasih saya karena saya membantu surat-suratnya, satu juta jadi 74 juta. Di kasih dari kampus x, memang dari kampus x itu saya dikasih sekitar 14 kalau ga salah, ohh 17 juta."

Code: Getting money
REC03

3.

"Jadi ada pekerjaan lagi terkait dengan pembebasan, pikirnya untung lah, lumayan untuk nambah-nambah uang dapur."

Code: Getting money
REC03

4.

"Jadi pikiranya yoo wes makelaran, oleh koyo, dikasih, diperseni iya sudah, selesai wes. Memang perjalanannya pada saat itu memang seperti itu aja. Jadi perjalanannya hanya seperti itu aja. Terus dikasih persen sama masyarakat yang sudah jual-jual, nganter dikasih ongkos, ngawal dikasih ongkos, cuman gitu-gitu aja wes. “Pak, Pak Carik tulung sampean terno, mbawa kendaraan sampean”. Iya sudah nganter ke Bank, dibayar di Bank. Dia bawa uang kes pulangnya, ngawal sampai rumahnya terus diamplopi, iya seperti itu."

Code: Getting money
REC03

5.

"I only got the fee for about 1 million, and also the sub-district head got 1 million. "

Code: Getting money
REC11 checked_translated 171104

6.

"So from that 8 spots, maybe I only took 10 million. 800 [million] was taken by Mr. x. Then it was shared within the provincial government, to the treasury director, to the council, those were the ones who approved the proposal."

Code: Getting money
REC11 checked_translated 171104

7.

"The Almighty was that I only received 1000 [one million]."

Code: Getting money
REC11 checked_translated 171104

8.

"Mungkin saya dulu dipinjemi dengan dengan imbalan fee, fee bendera karena pinjam jasa rupa jasa, eeee…. Itu saya dikasih waktu itu 50 juta dari panitia. "

Code: Getting money
REC12

9.

"Dan setelah proyek itu selesai saya dikasih fee "

Code: Getting money
REC12

10.

"He’eh. Waktu itu. Kemudian setelah itu berjalan dengan baik, setelah selesai pencairan dan saya dapat fee"

Code: Getting money
REC12

11.

"Kenanya itu terima fee 50 juta yang saya ceritakan tadi, itu sudah termasuk mendapatkan to? "

Code: Getting money
REC12

12.

"Pak H, transfer balik ke Pak B 70%". Dia yang kasih rekening saya. Saya ndak kenal pak B, "Siapa pak B?". "Pokoknya transfer ke sana 70%". "Nah 70% ini ke mana?", saya bilang. "Bawa ke saya sekian persen, Pak H pakai sekian persen, sekian persen untuk lembaga""

Code: Getting money
REC16

13.

"Pikiranku tadi, saya ikut pak E, paling saya dapat sertifikat ya, atau dapat transport makan"

Code: Getting money
REC16

14.

"And I didn't use it at all. Even if I got some, it was still from the operational expenses that were used. For example [I] used x percent, there was some operational fees for the officer"

Code: Getting money
REC33 checked_translated 171114

15.

" Itu berupa gaji yang masuk ke ibu sendiri?
(It went into your salary?)
P: Ya, ya semua, satu kantor termasuk sekretaris dan bendahara.
(Yes, yes [for] all, the whole office including the secretary and treasurer.)
R: Itu untuk kesejahteran?
(That was for the welfare?)
P: Iya, untuk kesejahteraan semua.
(Yes, for common welfare.)"

Code: Getting money
REC33 checked_translated 171114

16.

"Well, this is what I was thinking. At that time, I thought if this grew rapidly, automatically besides from salary, say, there were lots of fees from the economy sector, that's what I was thinking. There were a lot of operational fees from economy sector, at the beginning, when it was still in its infancy, it depended on the community forum. But, when it grew larger, it depended on the regulations that were made. In 2000, there wasn't any regulation that stated, say, a certain percentage from economy sector should be allocated for social or for the institution, or for the capital. There wasn't any. But after it grew, there was an operational and technical procedure which stated so. Then when we started to enjoy it, the regulations came. So, a percentage should be allocated for the capital, and some for the institution. And for the institution, it was also divided, e.g. for quality assurance, infrastructure, and others. So certain amount was for employee salary, for the infrastructure support, for renovation. So it wasn't purely, say, after the rapid growth, it wasn't depended on me, it's not that I got the most of it, nope. If it grew bigger, there were some rules, this much was for the chairperson, for the treasurer and so on... It existed. Undoubtly there were some for the chairman or the highest ranking officer. So, in my office, I still was the highest"

Code: Getting money
REC33 checked_translated 171114

17.

"Akhirnya pada satu kesepakatan dengan apa ini, sampai ketingkat Jasa, dapat fee, sampai di situ terbitkan surat kuasa."

Code: Getting money
REC36

18.

"Kalo kita bangun pasar dari swasta kita dapet base income, bukan dari pasar tapi dari mereka. "

Code: Getting money
REC38

19.

"Kedua dari aspek finance, kita otomatis dapet intensif juga dari sana juga, walikota"

Code: Getting money
REC38

2.1.8. Getting the profit

1.

Code: Getting the profit
REC37

2.2. Personal gain (self-esteem)

2.2.1. Showing ability

1.

"The point is, what I wanted was to get the acknowledgement that “I was able to do it”, to do a good job. That was it. So that I was able to get the fundings every year, to get the fundings again, that was my obsession."

Code: Showing ability
REC32_translated_checked

2.

"It has its’ own satisfaction, well maybe, that’s what people say as arrogant. It used to be my obsession, to show that I could."

Code: Showing ability
REC32_translated_checked

2.2.2. Pride

1.

"Dan mengangkat derajat, punya kemampuan ekonomi yang lebih dari pada teman-teman seangkatan. Mengangkat derajat, itu juga. Iya banyak duitnya, dari hasil korupsi, hehe! Meraka ga tahu bahwa kita korupsi."

Code: Pride
REC01

2.

"Seakan-akan kesobongan dirinya sendiri “ini, saya loo pegang uang” padahal uang-uang bahaya, ini kan, ga terasanya di situ"

Code: Pride
REC04

3.

"Terus timbul diluar itu, namanya masih muda, iya kan? Imannya kurang, terus gengsi-gengsian. Sama, sesame lurah atau sesame teman ini, sudah dipanggil Pak lurah masak ga nduwe duek. Ga terasa kita ngobrol apa-apa, mbayar kopi, mbayar apa. Wong di luar itu

banyak sekali, ada yang mau nyanyi, ada yang mau ini. Terus gengsi-gengsian ada pas hajatan kita nyawer atau apa, kan ndak ada pertimbangan yang penting punya uang."

Code: Pride
REC04

4.

"Hanya waktu itu hanya gengsi, pas ndak ada uang, kok pegang uang, hehe! Uang panas ini! Hehehe! Berarti ga mikir uang panas ini, dipake satu juta “Ahh! Besok dapat uang” lah ini loo tak ganti, padahal buat ganti ga ada."

Code: Pride
REC04

5.

"Malah ada kebanggan proposal saya diterima, tapi dananya dipotong setengah malah. Kita kemana-mana. Mereka tinggal ongkang-ongkong"

Code: Pride
REC19

6.

Code: Pride
REC22

7.

"Meminta waktu tambahan pun saya gak pernah, karena eee… Di lingkungan kebetulan saya banyak orang yang menghutang-hutangkan uang dengan sistem seperti itu dan saya juga pegawai negeri, jadi saya sungkan kalau sampe tidak tepat waktu"

Code: Pride
REC27

8.

"Tapi saya tidak tahu. Saya yang tahu hanya malu, saya gak mungkin tidak membayar apalagi tetangga-tetangga saya. Akhirnya seperti itu terus. Kalau menimbang ketika saya memalsukan mungkin saya jauh dari perilaku itu sehingga saya seperti ini. "

Code: Pride
REC27

9.

"Ke orang… ke pribadinya misalnya bapak menagih saya, saya gak bisa bilang, “Aduh! P saya gak bisa bilang gak punya uang.” Saya gak bisa bilang itu. Awalnya saya hanya saya harus punya uang ketika jatuh tempo saya harus bisa membayar. Ketika saya pinjam lagi, saya bisa dipercaya, gitu. Kan ketika eee… cicilan bank itu di ujung bulan misalnya tanggal 30/31, saya harus menyediakan uang"

Code: Pride
REC27

2.2.3. Being appreciated

1.

"Bukan kok punya rencana uang saya korupsi ini tak buat seperti ini, ndak ada rencana seperti itu. kebutuhan sesaat dan mendadak, kalau dibilang untuk, apa iya, rasa wah dan butuh sanjungan."

Code: Being appreciated
REC04

2.

Code: Being appreciated
REC14

3.

"Nggak saya, saya senang dengan hasil penelitian saya diapresiasi. Saya belum pernah gini, malah saya bercita2 hilangkan cuka dari pabrik tahu, cuka itu berbahaya untuk perut, gigi. Kan ada peneliti seperti Kuri untuk si radio aktif . Dia mati karen penelitianya, saya malah masuk penjara ya gapapa. Saya juga mengembangkan ini, saya berkorban dalam hal itu. Ada kepuasan"

Code: Being appreciated
REC19

4.

"Hasil penilaian itu tadi, menciptakan jiwa wirausaha dan inovasi baru memang kesenangan dan hobi kita. Dan dibiyai. Dan hasil penelitian kita diapresiasi. Gag tahu orang memanfaatkan ternyata. Kita kan bukan orang politik tapi scientist bener-bener. Tuhan semua yang ngatur. Memang intinya kita senang diapresiasi namun secara tidak sadar kita dimanfaatkan orang. Mungkin lain kali saya harus berhati-hati. "

Code: Being appreciated
REC19

5.

"We had good people from the district. The district was able to manage the funding, that was the main aim. What was bad from the village, we tried to conceal it. Even though there was a little bad creadits, we had to conceal it."

Code: Being appreciated
REC32_translated_checked

2.2.4. Being popular and remembered

1.

"Maybe at that time... for my name. You know, if my village was renowned because of the project, “the sub-district head wasn’t even able to get the project, can you imagine that a nobody, a villager like me, was able to get the project. Imagine that...” “Oh he knew Mr. x [the governor]”. You know, people will think like that.)"

Code: Being popular and remembered
REC11 checked_translated 171104

2.

" Yea, and to get noted as well. But, it was only, so that people will remember. And I got in here, my struggle and misery would be remembered by my people."

Code: Being popular and remembered
REC11 checked_translated 171104

2.3. Personal gain (job)

2.3.1. Doing the jobs description

1.

"Karena wong tugasnya TU itu ya ngetik sama bikin surat itu. Kan kalau bikin surat itu kita bikin, deal, kita print, kita ajukan ke pimpinan, kalau yang keliru dicoreti, kita ketik lagi, masih ada yang keliru dicoreti lagi, kita ketik lagi, dah selesai kita arsip. "

Code: Doing the jobs description
REC15

2.

"Karena itu tugas saya juga, ya saya harus mengetik"

Code: Doing the jobs description
REC15

3.

"Karena memang tugas saya sebagai bawahan job descriptionnya saya itu ya ngetik itu sama ngarsip surat"

Code: Doing the jobs description
REC15

2.3.2. Job security

1.

Code: Job security
REC29B

2.3.3. I wanted my company to be a big company

1.

"Mau dapat proyek besar. Jujur itu, makanya saya bilang nafsu tadi. Orang ga boleh serakah. Saya dapat segitu mas, rezeki itu selalu ada. Proyek kecil-kecil yang diselesaikan, cukup gaji karyawan, menghidupi keluarga sudah cukup. "

Code: I wanted my company to be a big company
REC36

2.3.4. Career development

1.

"Saya dulu tertarik diajak adik kelas yang di universitas x itu ini karena bernuansa pengabdian masyarakat. Saya butuh satu tingkat dari kegiatan ini. Paling tidak untuk akreditasi golonganku, gitu lho. PNS, yyayaya. "

Code: Career development
REC16

2.

"Oh Pak H dapat sertifikat hari ini satu, besok satu". Itu kan buat poin golongan kepangkatan kita kan. Dulu pikirku sederhana gitu"

Code: Career development
REC16

3.

"Jadi ada pengabdian masyarakat. kan tridarma di situ."

Code: Career development
REC16

4.

"Jadi kan kita mengurus dalam kredit point harus lengkap, pendidikan, pengaajaran, penelitian, pengabidan masyarakat. Nah itu, selain berkelakuan baik ya, itu menjadi pertimbangan Dekan untuk pengurus DP3 (Daftar penilaian Pegawai Negeri) kan. Tiap kali ngurus kredit poin, pangkat dibawa ke x. "

Code: Career development
REC16

5.

"Ya itu tadi secara akademis di kampus kana ada tri darma, pengabdian masyarakat dapet sertifikat. Sebagai pemateri poinnya sekitan. sebagai panitia dapat poin sekian. Kalo internasional sekian. Itu secara pribadi butuh "

Code: Career development
REC16

6.

"Prestasi kan dapet otomotasi di ujung. Itu kan tidak mengganggu PD pasar satu rupiahpun. Sudah dapet bangunan, base income, prestasi."

Code: Career development
REC38

2.3.5. Getting the jobs/projects

1.

"Kalau ndak nurut saya nggak dikasih kerjaan dong"

Code: Getting the jobs/projects
REC16

2.

"My intention was let’s say I get support from the district head, get the mission and get support."

Code: Getting the jobs/projects
REC32_translated_checked

3.

" Saya naluri bisnis pada waktu itu orientasinya saya dijanjikan kalau nanti nanti tahun depan ada perencanaan “nanti proyek itu bisa kita berikan untuk mas E langsung. "

Code: Getting the jobs/projects
REC36

4.

" Ga ada, kalau fee nomor sekian aja bagi saya. Saya yang dijanjikan pekerjaan besar itu. "

Code: Getting the jobs/projects
REC36

5.

"Mau dapat proyek besar. Jujur itu, makanya saya bilang nafsu tadi. Orang ga boleh serakah. Saya dapat segitu mas, rezeki itu selalu ada. Proyek kecil-kecil yang diselesaikan, cukup gaji karyawan, menghidupi keluarga sudah cukup. "

Code: Getting the jobs/projects
REC36

2.3.6. For my company

1.

Code: For my company
REC28

2.4. Society/Community

1.
"Dari dulu saya sudah memperhatikan masalah wawasan nusantara yang menurut saya sangat perlu ditanamkan pada generasi muda. Saya sudah sering melaksanakan kegiatan seperti ini bersama komunitas saya. Karena saya itu memang merasa terpanggil untuk melakukan itu. "

Code: Society/Community
REC05

2.
": Ya itu tadi mas, saya kan merasa terpanggil untuk melakukan kegiatan sosialisasi ini. "

Code: Society/Community
REC05

2.4.1. Helping friend

1.
"Jadi dalam kasus saya, saya hanyalah seseorang yang diminta bantuan oleh teman yang kebetulan teman itu adalah yang mempunyai jabatan di suatu institusi, perguruan tinggi swasta yang kemudian meminta tolong kepada saya apakah saya mempunyai teman yang mempunyai perusahaan dengan spesifikasi tertentu. Saat itu saya mengatakan kalo saya sendiri nggak ada pak."

Code: Helping friend
REC07

2.
"Yang perlu digarisbawahi bahwa saya memang sudah beberapa kali terlibat kerja sama dengan perguruan tinggi ini secara pribadi. Dalam kerjaan saya yang seperti itu dan teman saya mempercayakan beberapa pekerjaan kepada saya tapi untuk pekerjaan yang ini, saya diminta hanya untuk memperkenalkan gitu, sehingga perguruan tinggi ini dapat melakukan suatu proyek. "

Code: Helping friend
REC07

3.
"Artinya, disini bahwa saya berpikir pada saat itu saya hanya menolong seorang teman, setelah itu terus berlanjut teman ini minta data,data pekerjaan ini. Masing-masing perusahaan itu diminta penawaran,sudah dilakukakan, sudah dikerjakan"

Code: Helping friend
REC07

4.
"Jadi pada saat itu pikirnya membantu teman?
P: iya, hanya begitu karena pada saat itu pikiran apapun dengan nilai proyek yang ada sekitar 2,4 M itu ya."

Code: Helping friend
REC07

5.
": He’em. Membantulah, maksud saya begitu membantu. Hanya membantu saja gak berpikir jauh lebih dari itu. Gitu lho. "

Code: Helping friend
REC12

6.

"Jadi, istilahnya saya membantu mereka temen-temen saya. Nah kebetulan yang saya bantu itu kreditnya macet. Tidak membayar mereka dan akhirnya saya bertanggung jawab. "

Code: Helping friend
REC25

7.

" iya Cuma ingin bantu temen.niat kita baik tapi jadinya seperti ini. "

Code: Helping friend
REC25

8.

"Masalahnya saya cuma membantu teman tapi ternyta teman saya seperti itu. Jadi, yang kena tetep saya"

Code: Helping friend
REC25

9.

"membela teman, sampai akhirnya saya yang masuk"

Code: Helping friend
REC25

2.4.2. Helping the farmers

1.

"iya saya sosial. Saya itu gag ada yang gaji. Malah dengan program itu saya keluar uang sendiri. Petani saya ajari bikin pupuk sendiri, petani saya ajari birokrasi gini"

Code: Helping the farmers
REC09

2.

"intinya ingin meningkatkan kualitas produksi pertanian dan ini salah satu program apabila kita jalankan dg benar, ini jalan. Artinya yg gag benernya sarana produksi yg diberikan tidak berkualitas akhirnya "

Code: Helping the farmers
REC09

3.

"satu semangat kami bahwa kami ingi petani mendapatkan pendapatan yang lebih dengan peningkatan kualitas produksi dan yang kedua saya lihat bunganya ringan (0.5 %) .Jadi petani kita juga tidak keberatan. Ya itu aja, daripada kita pinjem ke bank"

Code: Helping the farmers
REC09

4.

Code: Helping the farmers
REC26

2.4.3. My village get funding again

1.

"I only wanted to get the fund back, so the village gets the fund as well, that was my intention, eventhought maybe I went through the wrong way. That’s why, have to be more careful now if working with other people."

Code: My village get funding again
REC32_translated_checked

2.
"That was it. So that I was able to get the fundings every year, to get the fundings again, that was my obsession."
Code: My village get funding again
REC32_translated_checked

3.
"And to get the fundings again."
Code: My village get funding again
REC32_translated_checked

2.4.4. For the public facility

1.
"I was just a villager, you know. Eee... I just wanted to get funding for the construction. Certain amount was corrupted, well it was on the other party"
Code: For the public facility
REC11 checked_translated 171104

2.
"What I truly want was to make the road comfortable. [So] Car can pass through. That's true. Well that's how it went, that's why I'm telling this. The locals can check this, and the road was checked as well"
Code: For the public facility
REC11 checked_translated 171104

3.
"I [did it] truly for the sake of my village, [so] it's not how it used to be. So that relatives, family, where ever they come, they can drive their car up to our yard. "
Code: For the public facility
REC11 checked_translated 171104

4.
"Me... My main consideration was if I go out and in to the village, the road is comfortable. And for the neighbors, so that everything there [what they sell], especially the farming products, the price could lift up a little bit."
Code: For the public facility
REC11 checked_translated 171104

5.
"Aha, yes. I was thinking about how to make it smooth. "
Code: For the public facility
REC11 checked_translated 171104

6.
"Multi effectnya kalo bandara ada, kan untuk masyarakat saya."
Code: For the public facility
REC24

7.

Code: For the public facility
REC30A

2.4.5. For the people

1.
" I struggled for my people. I was not a civil servant or anything, I was just a local figure"
Code: For the people
REC11 checked_translated 171104

2.
" I thought, “Me and the people got the grant, we struggled for the construction, for the road, not for myself or my family, not for it, [but] for all of us"
Code: For the people
REC11 checked_translated 171104

3.
"Even though the attorney went to my house 4 or 5 times. He knew my condition, my family, and all. Maybe he also knew my wealth. Well, I thought I was only working for the people. S"
Code: For the people
REC11 checked_translated 171104

4.
Code: For the people
REC22

5.
Code: For the people
REC22

6.
Code: For the people
REC22

7.
Code: For the people
REC22

8.
"Multi effectnya kalo bandara ada, kan untuk masyarakat saya."
Code: For the people
REC24

9.
Code: For the people
REC30A

2.4.6. My village

1.

"Well, because I was working at that time. They asked me to work there. If there is builded [university], my village could develop well, improving the living standards, public welfare, if it's [the building was] being built, the number of students will increase. That was my hope for the future. I didn't know that I was signing the document for the disbursement of the money, I didn't know that. I thought it was just to represent one of the land sellers. I didn't know that as a matter of fact it was used for the disbursement. During the legal proceedings, I realized that I was representing the whole 70 people, I just knew it then."

Code: My village
REC02_translated_checked

2.

"I only wanted to do good, but it went the other way! I didn't have any bad intention, I only wanted to help; if the buildings [university] are built then my village will be improved."

Code: My village
REC02_translated_checked

3.

"So when I came back I thought "why there's no development". Aaah... That's why I came back [to the village], I mean, how to solve or to gain access towards the development of my village."

Code: My village
REC11 checked_translated 171104

4.

"The thing is that my village got the funding. I didn't think that I will get prosecuted."

Code: My village
REC11 checked_translated 171104

5.

"I [did it] truly for the sake of my village, [so] it's not how it used to be. So that relatives, family, where ever they come, they can drive their car up to our yard. "

Code: My village
REC11 checked_translated 171104

6.

"Me... My main consideration was if I go out and in to the village, the road is comfortable. And for the neighbors, so that everything there [what they sell], especially the farming products, the price could lift up a little bit."

Code: My village
REC11 checked_translated 171104

7.

" I only worked for my village, so there was a development"

Code: My village
REC11 checked_translated 171104

2.5. Organization

1.

"But it was for the common good. For the school's progress. The funding was from the committee, the committee paid and took responsibility for it, and I had no problem with that. The latest project I had was land acquisition"

Code: Organization
REC06_translated_checked

2.

"Karena saya bawahan ya, terus pengen memajukan lembaga dan ada tnggung jawab terhadap lembaga. Ada informasi begini saya bawa ke Dekan. "

Code: Organization
REC16

3.

"Rewardnya waktu itu saya ini eee… untuk apa ini namanya, eee.., dana operasional itu ditambah."

Code: Organization
REC33 checked_translated 171114

4.

"The point is, even though the economy sector was huge, it was not for my own personal benefit, but for common purpose. So from that 205 million, when I develop it, it grew to billions. From that billion, a percentage was given back to the community. Even from the government regulation, a percentage should be allocated to social sector, for the institution development, and for the employees."

Code: Organization
REC33 checked_translated 171114

2.5.1. Supporting regional government project

1.

Code: Supporting regional government project
REC14

2.5.2. Organizational profit

1.

Code: Organizational profit
REC14

2.

Code: Organizational profit
REC17

3.

Code: Organizational profit
REC20

2.5.3. Organization reputation

1.

Code: Organization reputation
REC22

2.

Code: Organization reputation
REC22

3.

Code: Organization reputation
REC22

4.

Code: Organization reputation
REC22

5.

Code: Organization reputation
REC22

2.5.4. To build the market without spending any money

1.

" Kemudian ingin membangun pasar mandiri tanpa biaya APBD"

Code: To build the market without spending any money
REC38

2.

" Kita bisa banyak membangun pasar dengan dana pihak ketiga (CSR) tanpa menggunakan pribadi pak tanpa menggunakan dana pasar"

Code: To build the market without spending any money
REC38

2.5.5. Making money kept on growing

1.

" I was meant to make the money kept on coming. I allocated it to the villages that really need it. But here's where I was wrong, I knew I was wrong, but I thought it wasn't violating the PTU, the instruction manual, [the places] where it should be developed, I dared to do it, so I took the liberty. The money, as long as its... So [the budget] won't be freezed [idle]. "

Code: Making money kept on growing
REC33 checked_translated 171114

2.

" I thought, it actually was wrong, but as long as the funding still expanded, there won't be any issue"

Code: Making money kept on growing
REC33 checked_translated 171114

3.

"Well, the first one, so that the money will rapidly grow and not idle. Actually, that was my motivation."

Code: Making money kept on growing
REC33 checked_translated 171114

2.5.6. Employee's sake

1.
"Jadi, jujur saja, saya melakukan itu untuk kesejahteraan karyawan. Ketika komisaris, direksi, dll datang mereka enak-enak, sedangkan banyak orang kelaparan. "
Code: Employee's sake
REC21

2.
"saya bisa memberikan biaya operasional untuk menambah gaji staf itu juga ke bagian keuangan trus kedua saya ambil tagihan itu dan saya salurkan ke orang-orang tadi yang kelaparan."
Code: Employee's sake
REC21

3.
"Pada intinya saya melakukan ini karena ketidak adilan. Cuma salah saya, dan saya akui salah di sidang. Kita kerja tapi penghargaan apapun tidak kita dapatkan.
R"
Code: Employee's sake
REC21

4.
"Ya dibagi ketimbang diambil beliau yang gag ada kerjaanya. Banyak orang yang lebih berhak. UU juga menyatakan fakir miskin dilindungi negara. "
Code: Employee's sake
REC21

5.
"Uang saya ambil bukan untuk pribadi. Saya sama hakim dijuluki Robinhood kesiangan."
Code: Employee's sake
REC21

6.
"Ya, ya semua, satu kantor termasuk sekretaris dan bendahara.
(Yes, yes [for] all, the whole office including the secretary and treasurer.)"
Code: Employee's sake
REC33 checked_translated 171114

2.5.7. Promoting the organization

1.
"Ya itung-itung kita promosi lembaga lah ke mana-mana bawa banner kita", kan gitu,"
Code: Promoting the organization
REC16

2.
"Kita keliling bawa banner ke daerah-daerah. Spanduk kita dibawa sambil promosi,"
Code: Promoting the organization
REC16

2.5.8. To get the project (organization performance)

1.
"t was, in order to get the projects going.)"

Code: To get the project (organization performance)
REC06_translated_checked

2.

" I have to get one [dormitory] myself, and I think it’s very crucial. That’s why I think about the land issues. First of all, I had to have a land. That’s how it was."

Code: To get the project (organization performance)
REC06_translated_checked

3.

"Saya bilang, "Itu perintah yang punya uang Pak, kalau ndak begitu, saya ndak ada kegiatan kampus saya ini. "

Code: To get the project (organization performance)
REC16

4.

""supaya ada kegiatan pak. Kita keliling bawa banner ke daerah-daerah. Spanduk kita dibawa sambil promosi, sambil bagi-bagi brosur kita". Oh ya, masuk akal juga gitu kan."

Code: To get the project (organization performance)
REC16

5.

"Dan Dekan tertarik berminat mempromosikan ke daerah-daerah. Sudah jalan itu. Semua lembaga pemikiran seperti itu terutama kampus kecil yang kekurangan mahasiswa. Kan kalau ada kegiatan biasanya kan bawa banner, dan nyebar brosur dan satu desa tahu gitu. Tujuannya promosi kampus juga waktu itu, jadi setengah barter dengan kegiatan lah gitu. "

Code: To get the project (organization performance)
REC16

2.5.9. Organizational profit/income

1.

Code: Organizational profit/income
REC35

3. Information content

3.1. Open focus

3.1.1. Coordinating about do or don't do it

1.

"I always coordinate with the xxx Committee. Let's say "Sir, what if we are willing to pay 50-70 million?"

Code: Coordinating about do or don't do it
REC06_translated_checked

2.

"I've told my wife, "one day, either 2 or 3 more years I will caught myself into trouble". I told my wife that, "Before I get into trouble, want it or not, you have to give your consent. I plunged myself into it so that we have, like... oke, so that's fine if I got sentenced for 5 years, but we have our resourses (money)." I eventually told her that. Well it was rejected at the first time"

Code: Coordinating about do or don't do it
REC10_translated_checked

3.

"(Owh! I thought about it for so long, sir. Every night, almost every night I talked to my wife. About the same issue.)"

Code: Coordinating about do or don't do it
REC10_translated_checked

4.

" My wife said, "What if you don't take the money? You know, we still have our faith in God, maybe He will help us, because He knows that we are innocent." "Okay, God might help the ones who are innocent." "But in reality what was written in the documents and from the legal proofs, you are wrong. What would you say at the court? If you could find some loopholes to defend yourself, then do it. We have just enough by doing things rightfully." What's make it hard was that, sir. If I do it, worst case I got in, my financial is not compromised. If not, I will be suffering financially. But, can I actually defend myself? Can I found not guilty?"

Code: Coordinating about do or don't do it
REC10_translated_checked

5.

"I knew him [sub-district head] well, I asked him, "What do we do about this sir, [we] only received this much?"."

Code: Coordinating about do or don't do it
REC11 checked_translated 171104

6.

"Jadi saya bawa berita ini ke Dekan ku, katakan di kampus ku, Pak A kan Dekan. "Pak A, ada tawaran begini gimana, ambil nggak?"

Code: Coordinating about do or don't do it
REC16

7.

"Berbagai teman saya Tanya "gimana?", "Itu ambil aja, bagus itu. Saya dulu pernah dapat""

Code: Coordinating about do or don't do it

REC16

8.

"terus tanya ke kampus-kampus lain yang sering berkegiatan seperti itu. Semua menyetujui, "cuma sayang potongannya kebesaran pak H". Saya kan ngajar di ST,UP, diskusi sama lemlit-lemlit (kampus tersebut). Saya kan membimbing skripsi sudah lama. Jadi sama lemlit-lemlit kenal banyak. Di xx dengan senior-seniornya, di universitas X dengan lemlitnya. "

Code: Coordinating about do or don't do it
REC16

9.

"Iya, saya berunding dengan keluarga, berunding dengan keluarga. Kalau saya tetap bertahan, dia buka"

Code: Coordinating about do or don't do it
REC19

10.

"tapi saya berkoordinasi dengan bendahara saya menyetujui, saya jalan. Seandainya waktu itu, bendahara saya tidak menyetujui mungkin juga tidak akan terjadi seperti ini. Ya kan meski saya bendahar gaji, saya tetep bendahara pengeluar. Jadi, tidak mengambil keputusan sendiri"

Code: Coordinating about do or don't do it
REC25

11.

Code: Coordinating about do or don't do it
REC30B

12.

Code: Coordinating about do or don't do it
REC30B

3.1.2. Solution search/way

1.

"Finally, my relative came to my home. “please... since you’re an employee [a civil servant].)
R: Pegawai….
(Civil servant...)
P: “Golekno borongan khusus dalan iki. Samean lek rene yo penak, gak mlaku (carikan borongan khusus jalan ini. Kamu kalau ke sini ya enak, nggak jalan kaki)”, kan gitu.
(“Please try to propose a construction project for this road. So later if you come here, you don’t need to walk uphill.)
R: Hmmm…
(Hmmm...)
P: Akhirnya dikenalkan orang…, namanya Pak x.
(So he introduced me to Mr. x from x city.)"

Code: Solution search/way
REC11 checked_translated 171104

2.

"kita juga minta kirim surat ke pajak, ke provinsi. Tidak diberikan jawaban."

Code: Solution search/way

REC24

3.
"iya , kira sudah ngirim surat resmi pak ke kantor pajak untuk mereka mengatakan bahwa mereka tidak boleh jadi tim penafsir. Trus akhirnya kita kirim ke provinsi, ke gubernur juga untuk minta petunjuk tapi dia nggak punya petunjuk juga"
Code: Solution search/way
REC24

4.

Code: Solution search/way
REC28

5.
" iya pengalaman sebelumnya dan kan networking sebelumnya. Sudah ada komunikasi"
Code: Solution search/way
REC38

3.1.3. Legal status

1.

Code: Legal status
REC14

2.
" Bagaimana?
R: Ke gubernuran x, provinsi Pak. Saya ke provinsi.
P: Untuk apa itu?
R: Konsultasi itu Pak.
P: Oh sempet ke situ juga?
R: Iya, konsultasi gimana ada gini gini gini gini? Gak iso Pak iki kudu nekakno wong propinsi harus opo? Pemaparan gitu istilahnya. Pemaparan harus dipaparkan nanti ini AMDALnya kan juga harus di, kan gitu.
P: He'em he'em he'em. Hmmm.
R: Sudah gak bisa. Wah tambah yakin saya ini. "
Code: Legal status
REC15

3.
" Ya. Sudah saya ke BPK, ke BPK saya. Sreeet. Gimana Pak ini saya ada gini gini gini gini..."
Code: Legal status
REC15

4.

Code: Legal status
REC18

5.
"Cuma pernah KPK waktu berdiri juga dulu. Kami pernah dikasih penyuluhan juga waktu itu. Kalo kita sifatnya bukan kita yang nerima, kita yang memberi kan gpp. Yang gag boleh kita sebagai aparat, kita yang nerima, kita yang kenak"

Code: Legal status
REC21

6.

Code: Legal status
REC22

7.
" Terus di perpres itu mengatakan bisa melakukan dengan musyawarah."
Code: Legal status
REC24

8.
"Akhirnya tetep melakukan musywarah mufakat aja. Itu kan juga diatur di kepresnya juga"
Code: Legal status
REC24

9.
"Saya sempat membaca karena teman saya bilang, gitu. Ketika saya sudah mulai terbuka eee… saya Tanya kepada teman saya setelah saya sudah mengakui kemana-mana kalau saya memalsukan data, saya Tanya teman saya yang memang mengambil jurusan hukum, "
Code: Legal status
REC27

10.

Code: Legal status
REC30B

11.

Code: Legal status
REC30B

12.

Code: Legal status
REC30B

13.

Code: Legal status
REC35

3.2. Corruption focus

3.2.1. Comparing (what others do)

3.2.1.1. Supervisor also did

1.
" kan ngak ada yang kena. Mungkin yang lebih besar dari saya banyak, direksi mungkin."

Code: Supervisor also did
REC21

2.
"Sekali lagi oknum yang dibawah melakukan itu karena di atasnya, istilahnya guru kencing berdiri, murid kencing berlari. Sepanjang guru kencing berdiri, jangan melarang murid kencing berlari. Tapi kalo diatas bener, dibawah sungkan. "
Code: Supervisor also did
REC21

3.2.1.2. Others behaviors were fine

1.
": itu tadi, kecewa, kecemburuan, trus faktor contoh yang terjadi tidak dibawa kesana ke ranah hukum"
Code: Others behaviors were fine
REC21

3.2.1.3. How others react in the same situation

1.
"owh iya mas,pastilah. ada yang menolak, tertarik. Menolaknya alasannya potongan terlalu besar jadi laporannya susah. Kalo kampusku kan kecil, satu fakultas, tertarik aja. Jadi terarik karena dekannya (bilang), "supaya ada kegiatan pak. Kita keliling bawa banner ke daerah-daerah. Spanduk kita dibawa sambil promosi, sambil bagi-bagi brosur kita". Oh ya, masuk akal juga gitu kan. Jadi pemikiran dekan itu betul juga. Yang nolak benar juga. kalau kampus besar ngapain ngerjain begituan kan. Kaya kampus I itu gak akan mau, pekerjanya banyak juga. Itu aja dulu itu. Andaikan saya mungkin paham kayak jaksa, ini bahaya, saya nggak mungkin ambil itu. Tanpa diskusi sama dekan tak akan saya ambil juga, tak akan tertarik."
Code: How others react in the same situation
REC16

3.2.1.4. It was common

1.
" Masalah potong memotong itu biasa mas!!. Itu sejak zaman dahulu kala. Jadi potongan-potongan itu, kadang kecil kadang besar, jadi potongannya aja yang beda, ada 30%, 10% itu kan biasa, biasa jahat mereka gitu loh"
Code: It was common
REC16

2.
"masalah potongan, kalo dana legal mereka sudah tahu, biasa mereka itu sudah biasa."
Code: It was common
REC16

3.2.1.5. Comparing the situation

1.
"My friends are, like, well there are a lot of them, so my friends who are also school principals, they have our own ways to get a project, they approached various parties"
Code: Comparing the situation
REC06_translated_checked

2.

"Madrasa X on B Street and S on T [Street] have [their own] dormitory. I had to have one as well. After all, my school is the biggest madrasa in sub-district M. I have to get one [dormitory] myself, and I think it's very crucial. That's why I think about the land issues. First of all, I had to have a land. That's how it was"

Code: Comparing the situation
REC06_translated_checked

3.

"hen I asked my friend, who was in the same position, "Does it occur in your district?", "never"."

Code: Comparing the situation
REC10_translated_checked

4.

"Yes I did. "How long have you managed that region?", he was with Kepanjen district which was larger than my district. "I've been here for 4 year, [I] never issued some money." That's where I had my suspicion"

Code: Comparing the situation
REC10_translated_checked

5.

" But my suspicion was why I'm the only one who was asked to issue money?)"

Code: Comparing the situation
REC10_translated_checked

6.

"Well, let's say it went well. From the economy worth of 250 million, now it had grew around... now billions in short amount of time. Comparing with my other friends, I was faster."

Code: Comparing the situation
REC33 checked_translated 171114

3.2.1.6. Others also did

1.

"Actually, almost everyone, other colleagues, do that as well. "

Code: Others also did
REC06_translated_checked

2.

"Oh okay. Well, there are some, I know about some other public madrasas. The other public madrasas do that as well. "

Code: Others also did
REC06_translated_checked

3.

": Info terkait apa itu?
(Information related to?)
R: Ya sama, sana pernah dapat proyek itu.
(Pretty much the same, the ones that got the project.)"

Code: Others also did
REC06_translated_checked

4.

": itu tadi, kecewa, kecemburuan, trus faktor contoh yang terjadi tidak dibawa kesana ke ranah hukum"

Code: Others also did
REC21

3.2.2. Legal consequence

1.

"Oh yes. Before that, I was, huh! I've read all the books about corruption, I read the news. "If I got arrested, I have to spend this much". Why all the corruptors, even after they were out from office, was able to do so and so. "Oh, like so..." I learnt all of it, sir. I don't want to be thrown a shovel after I hit the rock bottom, I don't want that. Hehehe. That's why."

Code: Legal consequence
REC10_translated_checked

2.

"Saya pikir, oh mungkin andai saya tidak bisa membayar saya pasti dipenjara, tapi pasti saya kena memalsukan dokumen dan saya baca di KUHP 6 tahun maksimal, ternyata saya masuk TIPIKOR. Hehehe. "

Code: Legal consequence
REC27

3.

"Lihat aja di 263 KUHP". Iya saya buka, ternyata ancaman hukuman. Ancamannya itu 6 tahun."

Code: Legal consequence
REC27

3.2.3. The way (how to)

1.

"Sebelumnya ga nyari-nyari info dulu?
P : Ndak, ndak wes, ndak. Jadi begitu dimintai bantuan, iya sudah bergerak. Jadi mulai pengumpulan data, data masyarakat itu iya, sampai nyari alamatnya, alamat orang-orang itu. Jadi kita bagi-bagi pekerjaan dengan temen-temen yang lain."

Code: The way (how to)
REC03

2.

" tadi daya katakan sebelum menangani punya ini saya sudah makelaran di mana-mana, gitu loo. Saya sudah membebaskan banyak puluhan hektar di mana-mana"

Code: The way (how to)
REC03

3.

"Apa saja yang dilakukan?
P: yang dilakukan apakah program ini bermanfaat bagi kita gag, trus pengembaliannya gimana, pupuknya apa? Itu sudah kami komunikasikan. Trus kita tawarkan kesana. Ow iya gpp. Petani mau dengan apa yang kita usulkan. Akhirnya terjadi itu. Kita tidak berpikir bahwa kita akan gagal panen"

Code: The way (how to)
REC09

3.2.3.1. Knowing the institution credibility

1.

"pasti informasi ini memang company ini mempunyai kapasitas,pun CSR. Kebetulan grup di hongkong punyanya sorrow, dibawah x, kemudian punya line bisninya apa aja, punya dana CSR atau kan masuk kematerian berapa nilainya, kita bergerak. "

Code: Knowing the institution credibility
REC38

3.2.3.2. Knowing creditor identity

1.

Code: Knowing creditor identity
REC17

2.

Code: Knowing creditor identity
REC20

3.

"No, because most of them knew me pretty well. Like, the village heads, we [I]'ve seen them during the general meeting, "this person has a certain character". I actually learn it. If their personality is...[bad], I won't give it to them, I wouldn't dare to"

Code: Knowing creditor identity
REC33 checked_translated 171114

3.2.3.3. How to mark-up

1.

"Yes, we came from the same district, hang out together. And if we for example wanted to mark-up some pricing, we share it"

Code: How to mark-up
REC32_translated_checked

3.2.3.4. How to do securely

1.

"Iya karena antisipasinya banyak. Sudah dipersiapkan semua. orang maling harus dipersiapkan dulu jalan lari kemana, haha! Kan begitu iya?"

Code: How to do securely
REC01

2.

"Sepeti ada kesempat kata bapak. Iya? Pada waktu kita melihat ada kesempatan itu sempat nyari-nyari info dulu ndak waktu itu?
P : Kalau info kan sudah banyak.
R : Biasanya dapat dari mana?
P : Info dari Koran kan sudah, antisipasinya kan begini, begini. Dari teaman "kamu begini, gimana?" teman pejabat "gimana kalau gini?" "ohhh, gini, gampang wes!" dia kata, haha!"

Code: How to do securely
REC01

3.

"kalau waktu itu informasi yang dicari itu apa Pak? Nyari info itu tepatnya tentang apa Pak?
P : Bagaimana antisipasi"

Code: How to do securely
REC01

4.

"Jadi waktu itu info yang dicari itu cenderung pada bagaimana caranya biar aman?
(So at that time, the information that you were looking for was how to get it safely?)
R: Iya, biar aman dan dana habis, itu ga boleh ada sisa. Kan gitu, kalau ada sisa desa kita dinyatakan sudah mampu dan kita ga dapat dana lagi.
(Yes, to secure it and to spend all the grant, there should be no leftovers."

Code: How to do securely
REC32_translated_checked

3.2.3.5. How to falsifying the report

1.

"Iya, kita belajar, kita kan kecamatan baru jadi, saya belajar dari kecamatan yang lama.
(Yeah, we learnt [from others], we were a rather new districht, I learnt it from older ones.)
P: Dalam rangka melakukan?
(In order to do it?)
R: Iya, itu sebagian, sebagian arus lingkungan, "jangan terlalu kalem istilahnya, jangan terlalu lugu, lurus gitu". Ini kan proyek dari pemerintah itu kan hibah", sudah saya pelajari."

Code: How to falsifying the report
REC32_translated_checked

3.2.3.6. How to safe my assets (from corruption)

1.

"(I've learnt, and I had my intention, so that my assets won't be confiscated. "Oh, it will be confiscated if the assets are registered by the name of such and such. Oh, so that's how it is."

Code: How to safe my assets (from corruption)
REC10_translated_checked

2.

"What I look for wasn't about law. It's more about how to secure my assets. I was only thinking about the future. In regards to the legal aspect, no matter how much it will take, let me face the suffering. So that my wife and child won't [suffer]... "

Code: How to safe my assets (from corruption)
REC10_translated_checked

3.2.3.7. The procedure for getting the loan

1.

Code: The procedure for getting the loan
REC28

2.

Code: The procedure for getting the loan
REC28

3.2.4. Safety

1.
"Waktu itu saya membandingkan dengan Kabupaten-kabupaten lain, x, x, terkait dengan kebijakan seperti ini. Saya lihat mereka tidak ada masalah ya."

Code: Safety
REC05

2.
": Ya pada waktu itu saya minta pertimbangan panitia, dan mereka bilang katanya tidak apa-apa. Ya berarti saya lanjut. "

Code: Safety
REC05

3.
"x pun bilang gpp. x sendiri pun sudah bilang, uang ini pun habis gpp. Kan CSR itu. Pengembalian juga dikembalikan separuh gag masalah karena gagal panen, keterangan gagal panen ada."

Code: Safety
REC09

4.
"Saya katakana ini gimana hmmmm untuk pengadaan aman gak?
P: Sempat mikir gitu ya?
R: Iya. Aman gak? maksudnya aman itu gak ada rekayasa dan lain sebagainya, maksudnya tender-tender wajar gitu lho. Wajar dan gak gak hmm. Maksudnya intinya gak ada manipulasi atau gimanalah yang tanda kutip. "

Code: Safety
REC12

5.
"Cuma yang ditanya itu sempet yang ya temennya itu tadi ya menanyakan tentang keamanan, gitu aja ya?
R: He'em. "

Code: Safety
REC12

6.
"ya, itu juga, aman tidaknya proyek ini. Banyak yang mengatakan aman, memang semua mengatakan aman ini kan program gubernur bukan abal-abal (SK gubernur). "

Code: Safety
REC16

7.

Code: Safety
REC28

8.

Code: Safety
REC28

9.

Code: Safety
REC28

10.

"Antara lain keselamatan itu. Si I kan menghubungi saya. Dia “Aku yang pasang badan”"

Code: Safety
REC36

11.

"Ada si, saya nanya kesalah satu teman. Iya, fine-fine aja. Bahkan teman saya berapa tahun lalu ikut bekerja di perusahaanya Pak S."

Code: Safety
REC36

4. Information source

4.1. Impersonal sources

4.1.1. Within organization

1.

"Apa saja yang dilakukan?
P: yang dilakukan apakah program ini bermanfaat bagi kita nggak, trus pengembaliannya gimana, pupuknya apa? Itu sudah kami komunikasikan. Trus kita tawarkan kesana. Ow iya gpp. Petani mau dengan apa yang kita usulkan. Akhirnya terjadi itu. Kita tidak berpikir bahwa kita akan gagal panen"

Code: Within organization
REC09

2.

" kan gag ada yang kena. Mungkin yang lebih besar dari saya banyak, direksi mungkin."

Code: Within organization
REC21

3.

": itu tadi, kecewa, kecemburuan, trus faktor contoh yang terjadi tidak dibawa kesana ke ranah hukum"

Code: Within organization
REC21

4.1.1.1. Supervisor/leader

1.

"Jadi saya bawa berita ini ke Dekan ku, katakan di kampus ku, Pak A kan Dekan. "Pak A, ada tawaran begini gimana, ambil nggak?"

Code: Supervisor/leader
REC16

2.

"jelas. Ya menurut saya sudah cukup banyak ya, pertama saya ke Dekan"

Code: Supervisor/leader
REC16

3.

"Sekali lagi oknum yang dibawah melakukan itu karena di atasnya, istilahnya guru kencing berdiri, murid kencing berlari. Sepanjang guru kencing berdiri, jangan melarang murid kencing berlari. Tapi kalo diatas bener, dibawah sungkan. "

Code: Supervisor/leader
REC21

4.

"tapi saya berkoordinasi dg bendahara saya menyetujui, saya jalan. Seandainya waktu itu, bendahara saya tidak menyetujui mungkin juga tidak akan terjadi seperti ini. Ya kan meski saya bendahar gaji, saya tetep bendahara pengeluar. Jadi, tidak mengambil keputusan sendiri"

Code: Supervisor/leader
REC25

5.

Code: Supervisor/leader
REC30B

4.1.1.2. Committee

1.

"R: Ya pada waktu itu saya minta pertimbangan panitia, dan mereka bilang katanya tidak apa-apa. Ya berarti saya lanjut. "

Code: Committee
REC05

2.

" I always coordinate with the DP3 Committee. Let's say “Sir, what if we are willing to pay 50-70 million"

Code: Committee
REC06_translated_checked

3.

Code: Committee
REC30B

4.1.1.3. Staff

1.

Code: Staff
REC18

4.1.2. Analyst report

1.

Code: Analyst report
REC17

2.

Code: Analyst report
REC20

4.1.3. Media

1.

"Oh yes. Before that, I was, huh! I've read all the books about corruption, I read the news. “If I got arrested, I have to spend this much”. Why all the corruptors, even after they were out from office, was able to do so and so. “Oh, like so...” I learnt all of it, sir. I don't want to be thrown a shovel after I hit the rock bottom, I don't want that. Hehehe. That's why."

Code: Media
REC10_translated_checked

4.1.4. Legal authority

1.

"Cuma pernah KPK waktu berdiri juga dulu. Kami pernah dikasih penyuluhan juga waktu itu. Kalo kita sifatnya bukan kita yang nerima, kita yang memberi kan gpp. Yang gag boleh kita sebagai aparat, kita yang nerima, kita yang kenak"

Code: Legal authority
REC21

4.1.4.1. Criminal law book

1.

"saya baca di KUHP 6 tahun maksimal, "

Code: Criminal law book
REC27

2.

"Lihat aja di 263 KUHP”. Iya saya buka, ternyata ancaman hukuman. Ancamannya itu 6 tahun."

Code: Criminal law book
REC27

4.1.4.2. The government regulation

1.

Code: The government regulation
REC22

2.

" Terus di perpres itu mengatakan bisa melakukan dengan musyawarah."

Code: The government regulation
REC24

3.

"Akhirnya tetep melakukan musywarah mufakat aja. Itu kan juga diatur di kepresnya juga"

Code: The government regulation
REC24

4.1.4.3. The audit board

1.

"Ya. Sudah saya ke BPK, ke BPK saya. Sreeet. Gimana Pak ini saya ada gini gini gini gini…"

Code: The audit board
REC15

4.1.4.4. The province government

1.

"Saya ke gubernuran Pak
P: Bagaimana?
R: Ke gubernuran x, provinsi Pak. Saya ke provinsi.
P: Untuk apa itu?
R: Konsultasi itu Pak.

P: Oh sempet ke situ juga?
R: Iya, konsultasi gimana ada gini gini gini gini? Gak iso Pak iki kudu nekakno wong propinsi harus opo? Pemaparan gitu istilahnya. Pemaparan harus dipaparkan nanti ini AMDALnya kan juga harus di, kan g"

Code: The province government
REC15

2.

" kita juga minta kirim surat ke pajak, ke provinsi. Tidak diberikan jawaban. Terus di perpres itu mengatakan bisa melakukan dengan musyawarah"

Code: The province government
REC24

3.

"iya , kira sudah ngirim surat resmi pak ke kantor pajak untuk mereka mengatakan bahwa mereka tidak boleh jadi tim penafsir. Trus akhirnya kita kirim ke provinsi, ke gubernur juga untuk minta petunjuk tapi dia gag punya petunjuk juga"

Code: The province government
REC24

4.1.4.5. Friend mayor in law

1.

"Saya sempat membaca karena teman saya bilang, gitu. Ketika saya sudah mulai terbuka eee… saya tanya kepada teman saya setelah saya sudah mengakui kemana-mana kalau saya memalsukan data, saya tanya teman saya yang memang mengambil jurusan hukum, “Ohiya itu mbak masuk dalam kasus pemalsuan dokumen. "

Code: Friend mayor in law
REC27

4.1.4.6. Sub district head

1.

"I knew him [sub-district head] well, I asked him, “What do we do about this sir, [we] only received this much?"

Code: Sub district head
REC11 checked_translated 171104

4.1.4.7. National land agency

1.

Code: National land agency
REC30B

4.1.4.8. Village land database book

1.

Code: Village land database book
REC30B

2.

Code: Village land database book
REC30B

4.1.4.9. The law division of regional government

1.

Code: The law division of regional government
REC35

4.2. Interpersonal sources

4.2.1. Self-knowledge/experiences

1.
"Sebelumnya ga nyari-nyari info dulu?
P : Ndak, ndak wes, ndak. Jadi begitu dimintai bantuan, iya sudah bergerak. Jadi mulai pengumpulan data, data masyarakat itu iya, sampai nyari alamatnya, alamat orang-orang itu. Jadi kita bagi-bagi pekerjaan dengan temen-temen yang lain."
Code: Self-knowledge/experiences
REC03

2.
" tadi daya katakan sebelum menangani punya ini saya sudah makelaran di mana-mana, gitu loo. Saya sudah membebaskan banyak puluhan hektar di mana-mana"
Code: Self-knowledge/experiences
REC03

3.

Code: Self-knowledge/experiences
REC14

4.

Code: Self-knowledge/experiences
REC22

5.
"No, I had experience working as an accountant. [I had] experience to apply it.)"
Code: Self-knowledge/experiences
REC32_translated_checked

6.
"Well I have working experience, that’s how. I worked in Lippo, Solo, that was my experience, as an accountant"
Code: Self-knowledge/experiences
REC32_translated_checked

7.
" iya pengalaman sebelumnya dan kan networking sebelumnya. Sudah ada komunikasi"
Code: Self-knowledge/experiences
REC38

8.

"pasti informasi ini memang company ini mempunyai kapasitas,pun CSR. Kebetulan grup di hongkong punyanya sorrow, dibawah x, kemudian punya line bisninya apa aja, punya dana CSR atau kan masuk khumaterian berapa nilainya, kita bergerak. "

Code: Self-knowledge/experiences
REC38

4.3. Intrapersonal sources

4.3.1. Colleague in the other organization

1.

"Sepeti ada kesempat kata bapak. Iya? Pada waktu kita melihat ada kesempatan itu sempat nyari-nyari info dulu ndak waktu itu?
P : Kalau info kan sudah banyak.
R : Biasanya dapat dari mana?
P : Info dari Koran kan sudah, antisipasinya kan begini, begini. Dari teaman "kamu begini, gimana?" teman pejabat "gimana kalau gini?" "ohhh, gini, gampang wes!" dia kata, haha!"

Code: Colleague in the other organization
REC01

2.

"Actually, almost everyone, other colleagues, do that as well. But the others have a clever team, Sir. Their subordinates were well skilled. Whereas me, I'm a fool, clumsy, that's why I'm here. It goes like that for the others as we"

Code: Colleague in the other organization
REC06_translated_checked

3.

"[I] went to the others [headmasters]. Colleagues whose appointed in other schools.)
P: Info terkait apa itu?
(Information related to?)
R: Ya sama, sana pernah dapat proyek itu.
(Pretty much the same, the ones that got the project.)"

Code: Colleague in the other organization
REC06_translated_checked

4.

"No, I didn't ask, not in the internal of x. In the internal of x..., the teachers understand nothing about it. I only asked the other headmasters. The teachers know nothing. They only know how to teach. Well, sometimes I get disagreements. But those who disagree [other headmasters], their school did not get any projects. They were truthful. The ones who were truthful are unharmed, but their madrasas haven't develop that much.)"

Code: Colleague in the other organization
REC06_translated_checked

5.

"xxx pun bilang gpp. xxx sendiri pun sudah bilang, uang ini pun habis gpp. Kan CSR itu. Pengembalian juga dikembalikan separuh gag masalah karena gagal panen, keterangan gagal panen ada."

Code: Colleague in the other organization
REC09

6.

"After one year in that district, the next year after that I began to suspect. “Why did they ask me to issue certain amount of money, even though it is supposed to be deposited to another account?” I was able to, spend the money, I did have the authority to do it. But their signature, their approval, you know. Then I asked my friend, who was in the same position, “Does it occur in your district?”, “never”."

Code: Colleague in the other organization
REC10_translated_checked

7.

"Yes I did. “How long have you managed that region?”, he was with x district which was larger than my district. “I’ve been here for 4 year, [I] never issued some money.” That’s where I had my suspicion. When they asked me the same thing in the following year, I asked other person in other district. There were 30 districts inside Region M. My sub-district was the only one that were “asked” because of it’s tax potential, the villages is not that big, District S"

Code: Colleague in the other organization
REC10_translated_checked

8.

"Saya katakana ini gimana hmmmm untuk pengadaan aman gak?
P: Sempat mikir gitu ya?
R: Iya. Aman gak? maksudnya aman itu gak ada rekayasa dan lain sebagainya, maksudnya tender-tender wajar gitu lho. Wajar dan gak gak hmm. Maksudnya intinya gak ada manipulasi atau gimanalah yang tanda kutip. "

Code: Colleague in the other organization
REC12

9.

"Cuma yang ditanya itu sempet yang ya temennya itu tadi ya menanyakan tentang keamanan, gitu aja ya?
R: He’em. "

Code: Colleague in the other organization
REC12

10.

"Berbagai teman saya Tanya “gimana?”, "Itu ambil aja, bagus itu. Saya dulu pernah dapat""

Code: Colleague in the other organization
REC16

11.

"terus tanya ke kampus-kampus lain yang sering berkegiatan seperti itu. Semua menyetujui, "cuma sayang potongannya kebesaran pak H". Saya kan ngajar di ST,UP, diskusi sama lemlit-lemlit (kampus tersebut). Saya kan membimbing skripsi sudah lama. Jadi sama lemlit-lemlit kenal banyak. Di ST dengan senior-seniornya, di x dengan lemlitnya. "

Code: Colleague in the other organization
REC16

12.

Code: Colleague in the other organization
REC28

13.

Code: Colleague in the other organization
REC28

14.

Code: Colleague in the other organization
REC28

15.

Code: Colleague in the other organization
REC28

16.

Code: Colleague in the other organization
REC28

17.

Code: Colleague in the other organization
REC28

18.
"Yes, we came from the same district, hang out together. And if we for example wanted to mark-up some pricing, we share it"
Code: Colleague in the other organization
REC32_translated_checked

19.
"Jadi waktu itu memang, proses taunya waktu itu mulai dari?
(So at that time, the know how to started from?)
R: Dari kecamatan lain lah.
(From other district, of course.)
P: Ohh jadi kayak sering ngumpul-ngumpul?
(Ohh, such as hang out together?)
R: Iya jadi sering rapat, ngumpul-ngumpul gitu.
(Yes, meetings, hang outs.)
P: Waktu itu yang lebih difokuskan pada aspek apanya?
(You were focusing at which aspect?)
R"
Code: Colleague in the other organization
REC32_translated_checked

20.
"Well, let’s say it went well. From the economy worth of 250 million, now it had grew around... now billions in short amount of time. Comparing with my other friends, I was faster."
Code: Colleague in the other organization
REC33 checked_translated 171114

21.
"Antara lain keselamatan itu. Si I kan menghubungi saya. Dia “Aku yang pasang badan”"

Code: Colleague in the other organization
REC36

22.
"Ada si, saya nanya kesalah satu teman. Iya, fine-fine aja. Bahkan teman saya berapa tahun lalu ikut bekerja di perusahaanya Pak S."
Code: Colleague in the other organization
REC36

4.3.2. Informal relations

4.3.2.1. Family

1.
"Iya, saya berunding dengan keluarga, berunding dengan keluarga. Kalau saya tetap bertahan, dia buka"
Code: Family
REC19

4.3.2.2. Relative

1.
"Finally, my relative came to my home. “please... since you’re an employee [a civil servant].)
R: Pegawai….
(Civil servant...)
P: “Golekno borongan khusus dalan iki. Samean lek rene yo penak, gak mlaku (carikan borongan khusus jalan ini. Kamu kalau ke sini ya enak, nggak jalan kaki)”, kan gitu.
(“Please try to propose a construction project for this road. So later if you come here, you don’t need to walk uphill.)
R: Hmmm…
(Hmmm...)
P: Akhirnya dikenalkan orang…, n namanya Pak x.
(So he introduced me to Mr. x from x city.)"
Code: Relative
REC11 checked_translated 171104

4.3.2.3. Wife

1.
"I’ve told my wife, “one day, either 2 or 3 more years I will caught myself into trouble”. I told my wife that, “Before I get into trouble, want it or not, you have to give your consent. I plunged myself into it so that we have, like... oke, so that’s fine if I got sentenced for 5 years, but we have our resourses (money).” I eventually told her that. Well it was rejected at the first time"
Code: Wife
REC10_translated_checked

2.
"(Owh! I thought about it for so long, sir. Every night, almost every night I talked to my wife. About the same issue.)"
Code: Wife
REC10_translated_checked

5. Consideration/Evaluation

5.1. Push reasons

5.1.1. Rely on the others

5.1.1.1. Approved by the committee

1.

Code: Approved by the committee
REC22

2.

Code: Approved by the committee
REC22

3.

Code: Approved by the committee
REC22

4.

Code: Approved by the committee
REC30A

5.

Code: Approved by the committee
REC30B

5.1.1.2. Approved by the leader

1.
". Dana yang dianggarkan sekian tapi yang dikeluarkan sekian. Itu kan tidak sesuai. Tapi itu semua sudah ada nota kebijakan pimpinan untuk dilaksanakan meskipun tidak sesuai."
Code: Approved by the leader
REC05

2.
" Saya juga dulu mikirnya kalau tindakan yang saya lakukan sudah ada nota kebijakan dari pimpinan, sehingga saya berani. "
Code: Approved by the leader
REC05

3.
"Terus sama nota kebijakan dari pimpinan itu tadi sebagai landasan saya meskupin tidak sesuai kerangka anggaran. "
Code: Approved by the leader
REC05

4.

" Pertama, ke Dekanku ini. Mestinya Dekanku itu kena pak. Dekan itu yang menyetujui program ini apa tidak"

Code: Approved by the leader
REC16

5.

" Itu sepengetahuan dekan. Kalau dekan nggak ngizinkan, saya nggak akan ngerjain apa-apa loh!!. Saya kan bukan dekan."

Code: Approved by the leader
REC16

6.

"tapi saya berkoordinasi dg bendahara saya menyetujui, saya jalan. Seandainya waktu itu, bendahara saya tidak menyetujui mungkin juga tidak akan terjadi seperti ini. Ya kan meski saya bendahar gaji, saya tetep bendahara pengeluar. Jadi, tidak mengambil keputusan sendiri"

Code: Approved by the leader
REC25

7.

Code: Approved by the leader
REC30A

8.

Code: Approved by the leader
REC30B

9.

Code: Approved by the leader
REC34

10.

Code: Approved by the leader
REC34

11.

Code: Approved by the leader
REC34

12.

Code: Approved by the leader
REC35

13.

Code: Approved by the leader
REC35

14.

"Pengambil keputusan utamanya Dirut pak. Jadi saya dalam konteks diberikan mandatory oleh 3 direktur untuk menyelesaikan kebijakan-kebijakan itu di lapangan. Jadi tetep saya laporannya ke Dirut. Tapi memang dua kegiatan ini direksi sudah tahu dan setuju"
Code: Approved by the leader
REC38

5.1.1.3. I thought my supervisor had the responsibility on what I did

1.

"I thought he was being responsible related to this issue, but in the end, all of them stepped aside once we get investigated! Hehe"
Code: I thought my supervisor had the responsibility on what I did
REC02_translated_checked

2.

" I mean, he was responsible for the order, but he didn't take the responsibility for what he did; he gave the order, I did the signing. He didn't take his responsibility. "
Code: I thought my supervisor had the responsibility on what I did
REC02_translated_checked

3.

" The one who gave me order was the secretary, and he was my supervisor who supposed to be responsible about everything. I wasn't thinking of any legal issues. I didn't have any bad prejudice about it. "
Code: I thought my supervisor had the responsibility on what I did
REC02_translated_checked

4.

"(No, I thought that my supervisor had the responsibility on what I did."
Code: I thought my supervisor had the responsibility on what I did
REC02_translated_checked

5.

"saya sudah tahu itu salah saya sampaikan kepada orangnya , "Ini yang tanggung jawab sampean", gitu saya bilang. Dia bilang, "Ya siapa lagi yang tanggunng jawab kalau bukan saya?", itu. Karena secara lisan itu gak bisa ini Pak. Gak ada..."
Code: I thought my supervisor had the responsibility on what I did
REC15

6.

" Iya. Ya saya anggap oh dia yang bertanggung jawab. Ya kan? Karena saya sudah beranggapan gini, "Saya yang bertanggung jawab". "
Code: I thought my supervisor had the responsibility on what I did
REC15

7.

Code: I thought my supervisor had the responsibility on what I did
REC22

5.1.1.4. Trusted on the others

1.

"It didn’t come to my mind. I didn’t think that it’s a subject to corruption, not at all, because it was an Islamic school institution. I thought I was just giving a helping hand. If there’s any fortune, then I would gladly receive it, if not, that’s also fine. I thought it was an Islamic institution, so I don’t think it will... That’s what I thought."

Code: Trusted on the others
REC02_translated_checked

2.

" And it was an educational institution, I thought [they] were more detailed about what’s being processed, more meticulous and I thought there won’t be any problem. The committee consists of university’s lecturer, they were more meticulous, they should’ve known better. The checklist was based on the entry from the Group Requisite Definitive Planning committee. What I thought was, they were academics from an educational institution, so be it then. They wouldn’t deceive a villager like me, who had a good will. That’s what I was thinking, I"

Code: Trusted on the others
REC02_translated_checked

3.

"Cuma saya berpikir bahwa teman ini adalah seorang yang dapat dipercaya dan memang teman ini bisa saya percaya. Yang jadi masalah adalah kumpulan dari teman ini."

Code: Trusted on the others
REC07

4.

"ya karena kebetulan teman ini adalah teman yang kenal yang baik. Secara spiritual juga baik, itu pikir saya"

Code: Trusted on the others
REC07

5.

"Gag, kita menelaahnya dari BUMN mesti bagus trus harga yg diberikan kepada kami itu diatas masyarakat. Pikir kamu kualitasnya lebih bagus.ya, dia bilang kualitasnya lebih bagus dari pasaran. Harganya lebih mahal memang. "

Code: Trusted on the others
REC09

6.

" Saya pikir y biasa gak ada masalah. Wong dia juga biasanya mengerjakan itu. Gitu."

Code: Trusted on the others
REC12

7.

"Saya waktu itu karena ia sudah terbiasa pekerjaan seperti itu dan setiap tahun memang dia di banyak kabupaten yang lain juga mengerjakan dan gak ada masalah. Gitu. "

Code: Trusted on the others
REC12

8.

"Kan saya kan tahu mereka ya kredibilitasnya juga bagus di luar, gitu lho. Gak pernah ada masalah. "

Code: Trusted on the others

REC12

9.
": Jadi ya kayaknya santai saja memberi keputusan waktu itu karena saya tahu mereka lho yo. "

Code: Trusted on the others
REC12

10.
"Masalahnya tahu dan kenal. Kalau gak kenal gak anu gak mungkin mau, resiko juga. "

Code: Trusted on the others
REC12

11.

Code: Trusted on the others
REC17

12.

Code: Trusted on the others
REC18

13.

Code: Trusted on the others
REC18

14.

Code: Trusted on the others
REC18

15.

Code: Trusted on the others
REC18

16.

Code: Trusted on the others
REC18

17.

Code: Trusted on the others
REC18

18.

Code: Trusted on the others
REC18

19.

Code: Trusted on the others
REC23

20.

" percaya sama teman, membela teman, sampai akhirnya saya yang masuk. Tapi saya juga mengakui kesalahan saya karena terlalu gampang atau bodoh mengambil jalan seperti itu."

Code: Trusted on the others
REC25

21.

Code: Trusted on the others
REC28

22.

Code: Trusted on the others
REC28

23.

Code: Trusted on the others
REC34

24.

Code: Trusted on the others
REC34

25.

"Iya, saya percaya begitu saja. Berteman juga sudah lama dan ndak pernah saling melukai, iya to? Dan saya tau persis dia bergerak dibidang jasa konsultan. Baik konsultan rencana maupun superfisi dan cukup punya nama. Kemudian mengatakan yang mengerjakan di sana itu groupnya dia sendiri, jadi dia yang kerjakan pengawasan iya dia"

Code: Trusted on the others
REC36

26.

"Iya, awal-awal komunikasi lancar. Kan saya banyak diluar pulau. Lagian yang menjamin IS itu. Saya sudah bilang “Saya banyak kerjaan di luar lo, saya ga bisa ngontrol” “Aku yang tanggung jawab, ada apa-apanya aku”. Dia ikut masuk"

Code: Trusted on the others
REC36

27.

"tapi kan digaransi sama I suasana tadi itu, dan menjamin tidak akan terjadi itu. Saya pantau terus, Pak S sendiri yang menyatakan andaikan pekerjaan itu ndak selesai kita tetap jalan terus, kita selesaikan toh tinggal sedikit, sudah dapat izin dari bapaknya, tenang-tenang aja"

Code: Trusted on the others
REC36

28.

"pasti informasi ini memang company ini mempunyai kapasitas,pun CSR. Kebetulan grup di Hongkong punyanya x, dibawah x, kemudian punya line bisninya apa aja, punya dana CSR atau kan masuk khumaterian berapa nilainya, kita bergerak. "

Code: Trusted on the others
REC38

5.1.1.5. Trusted on supervisor

1.

"All of them [the documents] were filed by the village secretary, I was appointed by the secretary to sign it, so I didn't read it. I believed him. I assumed good faith from him as my supervisor; I didn't have any bad prejudice towards him or his order. In regards to the administration and the order, I had no bad prejudice. At the end, I didn't know that the paper that I signed was something that was invalid.)"

Code: Trusted on supervisor
REC02_translated_checked

2.

"They leave no clue at all, no basis, no background whatsoever. So I signed the document for the disbursement, to pay those sellers. At that time, I just comply with what was ordered"

Code: Trusted on supervisor
REC02_translated_checked

3.

"I thought, he was the one who did it, who would responsible about everything, but in the end it's not like that.)"

Code: Trusted on supervisor
REC02_translated_checked

4.

Code: Trusted on supervisor
REC23

5.

Code: Trusted on supervisor
REC23

5.1.2. That's the way it should be

1.

"Iya, karena itu interen tidak perlu, saya kan ada di dalam itu juga, iya to? Di interennya itu. Saya ngasihkan uang, karena uang itu kan butuh, iya to? Saya berikan, iya sudah laksanakan karena mereka bawahan saya. "

Code: That's the way it should be
REC01

5.1.2.1. There is no free lunch

1.

"Cuma kan gag ada yang gratis, artinya kita harus mancing dari depan dulu. "

Code: There is no free lunch

REC38

2.

"tetep kan gag ada yang gratis, ada negosiasi, entertain, kita bayar uang charge. Uang kita harus siapin di Bank itu trus, kalo gag ya gag bisa. Istilahnya kalo bapak mau pesen pesawat, kan bapak harus bangun landasan tuk turun. Nah landasannya biaya bapak. Jadi kan gag mungkin di kasih pesawat sekaligus landasannya. Jadi bapak sendiri yang harus siapin, pesawatnya turun, analoginya begitu. Tak ada yang gratis lah"

Code: There is no free lunch
REC38

5.1.2.2. It was impossible without giving back several percent from the

1.

"masalah potongan, kalo dana legal mereka sudah tahu, biasa mereka itu sudah biasa. Cuma potongnnya gede banget, itu aja. Jadi mereka itu paham, pemerintah di Indonesia itu, ya rata-rata emang begitu, tanpa potongan, gag mungkin"

Code: It was impossible without giving back several percent from the
REC16

5.1.2.3. In the crazy world, need to be crazy

1.

"At the end of the day I was like, “this is a crazy world we’re living in”, so “if we are not crazy enough, we won’t get anything”. Haha. In the end, that was the conclusion."

Code: In the crazy world, need to be crazy
REC06_translated_checked

2.

"First of all, that was the only way. As I said, this is a crazy world. That’s a Javanese saying. Zamanne zaman edan, lek gak melu edan ora keduman. Translated as “now, this is a crazy world we’re living in”, so “if we are not crazy enough, we won’t get anything. I"

Code: In the crazy world, need to be crazy
REC06_translated_checked

5.1.2.4. It was the best way

1.

"Want it or not, that was the best way, sir. Because I already went into the wrong way, wasn’t I?"

Code: It was the best way
REC10_translated_checked

2.

"Well, that, was what I capable of. [I thought] it was a good way. I don’t what this. But there was no other way, well... I got to stick with this."

Code: It was the best way
REC32_translated_checked

5.1.2.5. There was no other way

1.

"Yea, what I had in mind was I had to deal with it, whether I want it or not.)"

Code: There was no other way
REC06_translated_checked

2.

"That’s one consideration. First of all, that was the only way"

Code: There was no other way
REC06_translated_checked

3.

" I was just complying with all the process, to pay the fee. In reality, from 2010 to 2012 I didn’t get that project. After I complied, in 2013 they finally gave it to us. “Oh, that’s how it is"

Code: There was no other way
REC06_translated_checked

4.

"Want it or not, that was the best way, sir. Because I already went into the wrong way, wasn’t I?"

Code: There was no other way
REC10_translated_checked

5.

"Hmm. He’em he’em. Hmm. Berarti bisa dikatakan pada waktu itu gak ada pilihan?

P: Gak ada pilihan.

P: Selain melakukan itu?

R: Iya.

P: Hmmm."

Code: There was no other way
REC15

6.

"Karena wong tugasnya TU itu ya ngetik sama bikin surat itu. Kan kalau bikin surat itu kita bikin, deeel, kita print, kita ajukan ke pimpinan, kalau yang kliru dicoreti, kita ketik lagi, masih ada yang keliru dicoreti lagi, kita ketik lagi, dah selesai kita arsip. "

Code: There was no other way
REC15

7.

"Karena saya tidak ada pilihan lain, karena itu tugas saya juga, ya saya harus mengetik"

Code: There was no other way
REC15

8.

"kita pertimbangkan, kita juga minta kirim surat ke pajak, ke provinsi. Tidak diberikan jawaban."

Code: There was no other way
REC24

9.

"iya , kira sudah ngirim surat resmi pak ke kantor pajak untuk mereka mengatakan bahwa mereka tidak boleh jadi tim penafsir. Trus akhirnya kita kirim ke provinsi, ke gubernur juga untuk minta petunjuk tapi dia gag punya petunjuk juga"

Code: There was no other way
REC24

10.

"Karena kita bertiga jujur bukan dari keluarga yang wah ya, dari keluarga biasa-biasa, belum punya rumah, pegawai biasa gitu pak. Nggak seperti pejabat apa gitu, Cuma kan kalo minta

ke keluarga, jelas tidak punya kalo minta sebanyak itu. Yaa, tiba-tiba muncul ide seperti itu. Cari nasabah, pinjem nama, nanti uangnya buat bayar ini. Trus kalo setorannya sekian, harus cari nama berapa orang gitu. "

Code: There was no other way
REC25

11.

"iya, sudah paham kalo jalan yang saya lewati salah tapi berhubung tidak ada jalan lain, kasarannya saya sampek notok tidak menemukan. Jalannya Cuma satu itu dan akhirny yang membuat saya kecemplung. Jadi, kan saya harus ikhlas, legowo."

Code: There was no other way
REC25

12.

"Apa ya? Kepepet, terpaksa karena kondisi. "

Code: There was no other way
REC27

13.

"Artinya saya sudah sangat kepepet, sudah gak ada yang bisa saya bungakan lagi ke orang, baru saya nekat untuk itu.
P Dan menganggap itu cara terbaik?
R Iya, karena tidak ada cara lain dan tadi sudah saya sebutkan eeee… andai saya masih punya saudara lain yang bisa saya sambati saya gak akan seterburu-buru itu melakukan hal bodoh. "

Code: There was no other way
REC27

14.

"Well, that, was what I capable of. [I thought] it was a good way. I don't what this. But there was no other way, well... I got to stick with this."

Code: There was no other way
REC32_translated_checked

15.

"Kalau ga gitu kta ga dapat pekerjaan kedepanya."

Code: There was no other way
REC36

5.1.3. Doing the instruction

1.

" Jadi bertindak itu langsung karena perintah. Jadi sama perwakilan dari Pemkot itu diperintahkan "kamu kerjakan sana, sana, sana" iya wes bergerak sudah. Jadi bergerak, setiap bergerak itu tetap melalui ada perintah itu kalau ndak ada perintah iya sudah diam, ga ada kegiatan apa-apa, ada perintah baru bergerak lagi, begitu itu."

Code: Doing the instruction
REC03

2.

" Ya gimana mas ya, saya ini kan bawahan kalau tidak dilaksanakan nanti saya dibilang tidak taat pimpinan tapi kalau melakukan saya melanggar kerangka anggaran"

Code: Doing the instruction
REC05

3.

" Izin dekan, jadi bukan saya mengambil itu. Saya itu cuma disuruh kalau ada kegiatan, paling, "tolong dampingi kawan-kawan itu""

Code: Doing the instruction
REC16

4.
"Kalau ndak nurut saya nggak dikasih kerjaan dong""

Code: Doing the instruction
REC16

5.
"Yang saya lebih tertarik lagi ikut di situ itu karena ini, satu, perintah Dekan"

Code: Doing the instruction
REC16

6.
" tadi pertimbangan utamanya tetap ke Dekan. "

Code: Doing the instruction
REC16

7.
"Selagi dekan perintak kek gini,, ya kita nurut aja kan. Jadi pertimabngan kita, selain butuh kegiatan tridarma juga karena perintah Dekan. Saya pegawai yang disiplin tidak lepas dari izin dekan, pimpinan atau rektor."

Code: Doing the instruction
REC16

8.

Code: Doing the instruction
REC22

9.

Code: Doing the instruction
REC22

10.

Code: Doing the instruction
REC22

11.

Code: Doing the instruction
REC22

12.

Code: Doing the instruction
REC22

13.

Code: Doing the instruction
REC22

14.

Code: Doing the instruction
REC22

15.

Code: Doing the instruction
REC22

16.

Code: Doing the instruction
REC22

17.

Code: Doing the instruction
REC23

18.

Code: Doing the instruction
REC26

19.

Code: Doing the instruction
REC29B

20.

Code: Doing the instruction
REC29B

21.

Code: Doing the instruction
REC29B

22.

Code: Doing the instruction
REC34

5.1.4. Because I already went into the wrong way

1.

"My thoughts was, because I found myself in such situation, comparing to what I've issued earlier, I mean, how much I responsible for already, but I didn't get any contribution. The money was taken using my signature, with the forgery that I did, it was all my work but they took it. They never said, like "thank you, here's a little something to buy anything you want", no, nothing"

Code: Because I already went into the wrong way
REC10_translated_checked

2.

"So I just took it, like the Javanese says "Wes kadung teles nyemplung ae wes" [I got myself into it already, why don't I just go even further]. Right? You understand Javanese, right?"

Code: Because I already went into the wrong way
REC10_translated_checked

3.

"“Let's just do it all out, since I've became a criminal already", so I thought.)"

Code: Because I already went into the wrong way
REC10_translated_checked

4.

"Because I already went into the wrong way, wasn't I? Whether it was thoughoutly considered or not, it was wrong. It wasn't suppose to be done like that, wasn't it? But if I didn't do that, the aftermath would be worse! I let myself down. "

Code: Because I already went into the wrong way
REC10_translated_checked

5.

"I had to do it, because it was already being done. And to get the fundings again."

Code: Because I already went into the wrong way
REC32_translated_checked

5.2. Pull reasons

5.2.1. It was not corruption

5.2.1.1. Distrust with opposition colleague

1.

Code: Distrust with opposition colleague
REC13

2.

Code: Distrust with opposition colleague
REC13

5.2.1.2. The good decision

1.

Code: The good decision
REC14

5.2.1.3. Considering the behavior was the right way (make sense)

1.

Code: Considering the behavior was the right way (make sense)
REC18

2.

Code: Considering the behavior was the right way (make sense)
REC18

3.

Code: Considering the behavior was the right way (make sense)
REC18

5.2.1.4. Based on the rule/regulation

1.

Code: Based on the rule/regulation
REC17

2.

Code: Based on the rule/regulation
REC17

3.

Code: Based on the rule/regulation
REC20

4.

Code: Based on the rule/regulation
REC20

5.

Code: Based on the rule/regulation
REC20

6.

Code: Based on the rule/regulation
REC22

7.

Code: Based on the rule/regulation
REC26

8.

Code: Based on the rule/regulation
REC30B

9.

Code: Based on the rule/regulation
REC30B

10.

Code: Based on the rule/regulation
REC35

11.

Code: Based on the rule/regulation
REC37

12.

Code: Based on the rule/regulation
REC37

5.2.1.4.1. I didn't violate any regulation

1.

Code: I didn't violate any regulation
REC14

2.

Code: I didn't violate any regulation
REC20

3.

Code: I didn't violate any regulation
REC20

5.2.1.4.2. It was the legal land

1.

Code: It was the legal land
REC30B

2.

Code: It was the legal land
REC30B

5.2.1.4.3. It was auditable

1.

Code: It was auditable
REC26

2.

Code: It was auditable
REC26

5.2.1.5. It was the right way

1.

Code: It was the right way
REC14

2.

Code: It was the right way
REC22

3.

Code: It was the right way
REC22

4.

Code: It was the right way
REC22

5.

Code: It was the right way
REC22

6.

Code: It was the right way
REC26

7.

Code: It was the right way
REC31

8.

Code: It was the right way
REC31

5.2.1.6. *Didn't know corruption law*

1.
"kemudian saya tidak paham tentang undang-undang tentang itu, jadi undang-undang Tipikor, undang-undang untuk pelaksanaan pengadaan itu saya tidak tahu. Saya pikir waktu itu saya bukan panitia bukan pelaku."
Code: Didn't know corruption law
REC03

2.
" Andaikan saya mungkin paham kayak jaksa, ini bahaya, saya nggak mungkin ambil itu. Tanpa diskusi sama dekan tak akan saya ambil juga, tak akan tertarik. Secara waktu itu pemahaman hukum saya lemah. "
Code: Didn't know corruption law
REC16

5.2.1.7. *It was not corruption*

1.

Code: It was not corruption
REC13

2.
"Sederhana gitu, dan ini hibah. Hibah itu definisinya gitu itu, apa kata yang punya dana kan. Itupun nggak ngerti, definisi hibah dari mana. Taunya hibah yang diaudit juga. Nah, definisi hibah versinya Pak Eini, "ini aman""
Code: It was not corruption
REC16

3.
"Dulu kan saya gak mengerti akan ada masalah ini, karena saya denger ini hibah. Hibah itu pemberian aja lah seperti sampean ngasi ke saya, itu hibah kan. Saya sesederhana itu dapat defenisi hibah. Intinya saya kurang paham tentang tadi itu, pemahaman tindak pidanaa korupsi seperti apa. Saya nggak ngerti, blass gag ngerti. "
Code: It was not corruption
REC16

4.
"Cuma pernah KPK waktu berdiri juga dulu. Kami pernah dikasih penyuluhan juga waktu itu. Kalo kita sifatnya bukan kita yang nerima, kita yang memberi kan gpp. Yang gag boleh kita sebagai aparat, kita yang nerima, kita yang kenak. "
Code: It was not corruption
REC21

5.

Code: It was not corruption
REC22

6.

"Saya pikir, oh mungkin andai saya tidak bisa membayar saya pasti dipenjara, tapi pasti saya kena memalsukan dokumen dan saya baca di KUHP 6 tahun maksimal, ternyata saya masuk TIPIKOR. Hehehe. "

Code: It was not corruption
REC27

7.

"Makanya saya pernah ditanyai kawan-kawan pemkot yang namanya korupsi apa? “korupsi adalah ketika pengambil keputusan sudah mengambil seluruh fasilitas dia melakukan negosiasi lagi. Misalnya begini, saya sebagai pengambil keputusan setahun membangun pasar dengan budget 50 M. Jadi itu sudah ada feenya, tapi kalo masih ngambil fee yang lain itu yang namanya korupsi atau penggelapan. "

Code: It was not corruption
REC38

5.2.1.8. Considering the money was not for personal gain

1.

"What’s important for me is to have your approval. Let’s do it. If anything goes wrong, it’s a shared responsibility. As long as it’s not for our personal gain.”"

Code: Considering the money was not for personal gain
REC06_translated_checked

5.2.1.9. Did not receive the money

1.

" I thought if I don’t eat the money, I won’t get into jail just like that. I was like that. It was obvious who got them [the money], it was given to x, y, z... The receivers were obvious, everything was investigated. I did not receive any. That was my thought. "

Code: Did not receive the money
REC06_translated_checked

2.

"Well, I didn’t enjoy any of it. If there’s anything behind it I would say it already. Certain amount of money was given to this and that person. Later on all of them will be called.)
P: Hmm
(Hmm)
R: Gitu, saya kan tidak menerima sama sekali. Saya pikir, saya punya keyakinan seperti itu gitu lho.
(That’s how it is, I didn’t receive any of it. I have this kind of belief [to not receive anything].)"

Code: Did not receive the money
REC06_translated_checked

3.

"That’s the first reason. Secondly, the fact is I didn’t get any, right? Unless if I receive some, let say 50 out of 700.)"

Code: Did not receive the money
REC06_translated_checked

4.

Code: Did not receive the money
REC18

5.

Code: Did not receive the money
REC20

6.

Code: Did not receive the money
REC20

7.

Code: Did not receive the money
REC20

8.

Code: Did not receive the money
REC23

9.

Code: Did not receive the money
REC26

10.

Code: Did not receive the money
REC35

11.

Code: Did not receive the money
REC35

5.2.1.10. I didn't do any corruption

1.

"No, not at all. I plead, “I did’t do any corruption, Your Honor.” That was the only defence I can do, it was an order afterall, wasn’t it?"

Code: I didn't do any corruption
REC02_translated_checked

2.

"I was thinking, “they did the corruption, I didn’t do anything at all. I only got the fee for about 1 million, and also the sub-district head got 1 million."

Code: I didn't do any corruption

REC11 checked_translated 171104

3.

"Aha. The first thing I thought that, I told everyone that I didn't do any corruption. "The one who did it was those people". You know. "

Code: I didn't do any corruption
REC11 checked_translated 171104

4.

"The Almighty was that I only received 1000 [one million]. I didn't corrupt. It also includes my transportation fee, going here and there. "

Code: I didn't do any corruption
REC11 checked_translated 171104

5.

"(No, I wasn't thinking that far. Well I was a totally blind about law, I didn't even feel that I [did it], so be it."

Code: I didn't do any corruption
REC11 checked_translated 171104

6.

" I didn't think about it at all, if corruption came across my mind, why did I give it up? Better to take all the wealth for myself. A"

Code: I didn't do any corruption
REC11 checked_translated 171104

7.

"Well, I only think that, "I'm not the one who corrupt". That's what I was thinking. I only worked for my village, so there was a development. I didn't think that far."

Code: I didn't do any corruption
REC11 checked_translated 171104

8.

Code: I didn't do any corruption
REC35

5.2.1.11. I have no bad intention

1.

Code: I have no bad intention
REC14

2.

Code: I have no bad intention
REC31

3.

Code: I have no bad intention
REC34

5.2.2. Secure/safe

5.2.2.1. Committee said it would be safe

1.
"Ya pada waktu itu saya minta pertimbangan panitia, dan mereka bilang katanya tidak apa-apa. Ya berarti saya lanjut. "

Code: Committee said it would be safe
REC05

5.2.2.2. Others said that it would be safe

1.
"Ya pada waktu itu saya minta pertimbangan panitia, dan mereka bilang katanya tidak apa-apa. Ya berarti saya lanjut. "

Code: Others said that it would be safe
REC05

2.
"Karena pemilik mengatakan ya gpp asal clean. Clean disitu artinya asal pekerjaan itu memang diturunkan dan sesuai dengan perusahaan dan tidak menimbulkan permasalahan. Saya berusah seperti itu juga kepada pemilik proyek. Tetapi tidak tahunya ini yang terjadi, di belakang itu saya"

Code: Others said that it would be safe
REC07

3.
"xxx pun bilang gpp. xxx sendiri pun sudah bilang, uang ini pun habis gpp. Kan CSR itu. Pengembalian juga dikembalikan separuh gag masalah karena gagal panen, keterangan gagal panen ada."

Code: Others said that it would be safe
REC09

4.
"Banyak yang mengatakan aman, memang semua mengatakan aman ini kan program gubernur bukan abal-abal (SK gubernur). Yang saya lebih tertarik lagi ikut di situ itu karena ini"

Code: Others said that it would be safe
REC16

5.
"jelas. Ya menurut saya sudah cukup banyak ya, pertama saya ke Dekan terus tanya ke kampus-kampus lain yang sering berkegiatan seperti itu. Semua menyetujui, ""

Code: Others said that it would be safe
REC16

6.
"owh iya, pastikan pertimbangan-pertimbangan dekan mengatakan"oh ya aman pak H", jalankan. Jadi Kalo dekan bilang nggak aman, ya jangan."

Code: Others said that it would be safe
REC16

7.

Code: Others said that it would be safe

REC28

8.

Code: Others said that it would be safe
REC28

9.

Code: Others said that it would be safe
REC28

10.

Code: Others said that it would be safe
REC28

11.

Code: Others said that it would be safe
REC28

12.

Code: Others said that it would be safe
REC28

13.

Code: Others said that it would be safe
REC35

14.

Code: Others said that it would be safe
REC35

15.

"Iya karena I sendiri datang itu “ini proyeknya Pak Wali”. Saya bersedia karena ini aman. “Ini proyek Pak Wali saya sendiri yang ngawasi”. Terus yang namanya K dan S, S ini tanganya X, X dekat dengan Pak Wali."

Code: Others said that it would be safe
REC36

16.

"Iya, awal-awal komunikasi lancar. Kan saya banyak diluar pulau. Lagian yang menjamin x itu. Saya sudah bilang “Saya banyak kerjaan di luar lo, saya ga bisa ngontrol” “Aku yang tanggung jawab, ada apa-apanya aku”. Dia ikut masuk"

Code: Others said that it would be safe
REC36

5.2.2.3. Others' behaviors were fine

1.
"Waktu itu saya membandingkan dengan Kabupaten-kabupaten lain, x, x, terkait dengan kebijakan seperti ini. Saya lihat mereka tidak ada masalah ya. "

Code: Others' behaviors were fine
REC05

2.

Code: Others' behaviors were fine
REC20

3.
"Cuma selain saya agak marah. Sebelumnya banyak yang melakukan itu untuk pribadi tapi tak pernah masuk. Dan itu terucap langsung dari kepala cabang. "

Code: Others' behaviors were fine
REC21

4.
"Di perusahaan itu belum pernah ada yang masuk penjara. Dan direksi juga mengatakan nggak akan saya masukkan, kalo perbuat kesalahan. Itu yang mendorong saya."

Code: Others' behaviors were fine
REC21

5.
"kan gag ada yang kena. Mungkin yang lebih besar dari saya banyak, direksi mungkin. Tapi mereka bebas semua.bukan saya iri atau apa."

Code: Others' behaviors were fine
REC21

6.
"trus faktor contoh yang terjadi tidak dibawa kesana ke ranah hukum. Saya waktu keluar juga. "

Code: Others' behaviors were fine
REC21

7.

Code: Others' behaviors were fine
REC28

8.

Code: Others' behaviors were fine
REC28

5.2.2.4. My previous behaviors were fine

1.
"Sudah rutin seperti itu, 2010 iya begitu, 2009 iya begitu, 2008 iya begitu. Sudah biasa begitu aja."

Code: My previous behaviors were fine
REC01

2.

"ya kebetulan sebelum saya makelaran ikut orang-orang kampus U ini, saya sudah jauh-juah tahun itu saya sudah memang sering dimintai untuk pembebasan lahan. Seperti pabrik susu x yang di gunung x, 30 hektar waktu itu saya juga ikut terlibat di situ, ndak ada masalah. Terus pengadaan lahan, tanah untuk pembangunan kampus dua I saya juga terlibat di sana. "

Code: My previous behaviors were fine
REC03

3.

"Iya, ini aja kan tidak seberapa yang di kampus U ini. Yang bos saya di kota x aja tidak 10-20 hektar, ratusan. Saya yang di x itu 400 hektar saya ga ada masalah, ga ada masalah."

Code: My previous behaviors were fine
REC03

4.

" tadi daya katakan sebelum menangani punya ini saya sudah makelaran di mana-mana, gitu loo. Saya sudah membebaskan banyak puluhan hektar di mana-mana"

Code: My previous behaviors were fine
REC03

5.

"Dulu-dulu pernah tapi itu hanya satu dua kali, gak gak ada masalah. Kebetulan ini kan ada masalah politik juga juga, ada masalah bupati sama sekda, sekda juga kena satu kamar dengan saya di kota K. "

Code: My previous behaviors were fine
REC12

6.

"Kan biasa dulu dipakai bendera dipakai pendamping. Tahun-tahun segitu enak gak ada masalah"

Code: My previous behaviors were fine
REC12

7.

"Sebenarnya ya karena sebenarnya banyak orang meminjam, minjam-meminjam CV. Sepertinya dari mereka kan banyak pekerjaan yang di berapa kabupaten tempat itu seperti itu gitu lho. Jadi sering dia juga minjem-minjem. Bukan CVnya sendiri sih. Kan saya kan tahu mereka ya kredibilitasnya juga bagus di luar, gitu lho. Gak pernah ada masalah. Gak ngerti pas saya kok seperti itu. Jadi, gak mikir berat sih nggak. Gak ada pikiran kalau seperti ini sih gak ada sama sekali. "

Code: My previous behaviors were fine
REC12

8.

" Hee'em. Seakan-akan apa yang saya putuskan yang saya ambil sudah baik dan gak ada masalah karena selama ini juga gak ada masalah gitu. "

Code: My previous behaviors were fine
REC12

9.

Code: My previous behaviors were fine
REC17

10.

Code: My previous behaviors were fine
REC17

11.

Code: My previous behaviors were fine
REC20

12.

Code: My previous behaviors were fine
REC22

13.

Code: My previous behaviors were fine
REC29B

14.

Code: My previous behaviors were fine
REC31

15.

"And everything went well from, I did it since 2003. The first year there was no issue, the results were good. In 2004, 2005, 2006, 2007, 2008 there was no issue, in 2009 there's this election coming. Because I was in the politics, then things started to crumble."

Code: My previous behaviors were fine
REC33 checked_translated 171114

16.

"I was always got audited, got clarified, but they found nothing up until 2010, 2013."

Code: My previous behaviors were fine
REC33 checked_translated 171114

17.

"And it was because of the first, second, third, fourth year it was okay, there were no issues, even in 2007, I got the award, sir. So I was more encouraged to do so. So in the infrastructure aspect, I worked more on it."

Code: My previous behaviors were fine
REC33 checked_translated 171114

18.

"Eventually, oh well, I disbursed it but I was a bit afraid, but it went well. Oh okay then, I did it again and again. I eventually went on, but only if there was some idle money left, sir. If not, then I couldn't. We prioritize people [the eligibles]'s demands first"

Code: My previous behaviors were fine
REC33 checked_translated 171114

19.

Code: My previous behaviors were fine
REC37

20.

Code: My previous behaviors were fine
REC37

21.

"Jadi pertimbangannya karena saya pernah bekerjasama dengan mereka dan mereka pernah melakukan humanaterian project di Indonesia dan waktu saya bolak balik hongkong itu, saya pernah mengeluarkann transaksi pihak ketiga itu di swasta di China."

Code: My previous behaviors were fine
REC38

22.

"iya pengalaman sebelumnya dan kan networking sebelumnya. Sudah ada komunikasi. Kan saya juga pernah mengeluarkan dana hibah ke company saat saya di x, b sekitar sejumlah 10 juta hongkong=10 miliyar. Saya sebelumnya sudah pernah melakukan itu dan tak ada masalah. Karena swasta ke swasta gag ada masalah. Dan itu memang ada, dana comunity itu kan pasti ada 100% pasti ada apalagi S. Proposalnya keluar, sory ya, ita kan overliquid. Disamping dana untuk developing country, dan tujuan tertentu seperti politik, itu kan wajar. Selama tidak merusak."

Code: My previous behaviors were fine
REC38

23.

"saya karena sudah yakin dengan sebelumnya, makanya saya mengusulkan seperti itu ke direksi."

Code: My previous behaviors were fine
REC38

5.2.2.5. Considering the behavior will not make any issues

1.

" Jadi tidak terbesit sedikit pun kalau ini akhirnya jadi masalah, ga terbesit."

Code: Considering the behavior will not make any issues
REC03

2.

"Saya mengawalnya dengan seperti itu, saya tidak berpikir ada apa-apa dsb karena saya pikir proyek itu pekerjaan itu tidak terlepak dari kontrak spesifikasi yang ada. Saya hanya memastikan itu. Yang memang dalam istilahnya,dalam tanda kutip orang proyek biasa lakukan memang disitu ada terjadi seperti pinjem bendera. Nama perusahaan yang dijadikan pekerjaan itu. Itu yang terjadi memang. Itu saya tidak mempunyai bayangan sama sekali bahwa akan ada kerugian yang saya kemudian ternyata ada suatu hal yang ditutupi dari saya oleh kedua pihak itu"

Code: Considering the behavior will not make any issues
REC07

3.

" Sama sekali tidak punya pikiran apapun karena saya berpikir seperti tadi yang sampaikan. Kalo ada uang trus ada barangnya. Jadi pekerjaan itu memang dilakukan oleh kontraktor yang ditunjuk oleh perguruan tinggi itu untuk melakukan pekerjaannya dan dia pekerjaannya

sudah sesuai, ya sudah. Ternyata seperti itu tadi, ada double sumberdana. Kejaksaan melihat bahwa dana yang ditarik dari pemerintahan ini dan hibah itu. Itu tidak digunakan membangun"

Code: Considering the behavior will not make any issues
REC07

4.

" Cara saya itu tadi bahwa pekerjaan itu ada dan dikerjakan, ada barangnya. Itu yang membuat saya punya keyakinan bahwa pekerjaan itu bersih. Menurut pemikiran saya pada saat itu. Tapi setelah kejadian ini semua akan akan berhati hati dalam mengambil keputusan"

Code: Considering the behavior will not make any issues
REC07

5.

"Tapi gak sejauh itu mikirnya ya biasa. Gak berpikir nantinya seperti ini itu gak gak sama sekali. Ternyata ... "

Code: Considering the behavior will not make any issues
REC12

6.

"Lha saya gak berpikir kalau sampai jauh sampai gini, wong Bu Camat sudah mengeluarkan apa namanya? Pelepasan Hak atas tanah itu. Pelepasan Hak sudah dianu sama Bu Camat, ditandatangani Kepala Desa dst, pemilik tanah, dst."

Code: Considering the behavior will not make any issues
REC15

7.

Code: Considering the behavior will not make any issues
REC23

8.

"ya pertimbangannya waktu itu tak pernah menimbang akibatnya akan seperti ini. Cuma, yang saya tahu bagaimana menyelesaikan masalah ini. "

Code: Considering the behavior will not make any issues
REC25

9.

"nggak, jadi ya memang murni kita bertiga. Yang penting di kantor beres gitu lo. Kita nggak mikir akhirnya seperti ini"

Code: Considering the behavior will not make any issues
REC25

10.

"It wasn’t in my mind at the first time. We were “safe”."

Code: Considering the behavior will not make any issues
REC32_translated_checked

11.

"And in the other side, I was thinking, “well, that’s fine, as long as there’s no issue.” So, that’s what I thought. It’s fine as long as there are no issues. "

Code: Considering the behavior will not make any issues
REC33 checked_translated 171114

12.

"eventually I though, “okay, there won’t be any problem, its fine”. And everything went well from, I did it since 2003"

Code: Considering the behavior will not make any issues
REC33 checked_translated 171114

13.

"So I didn’t thought that the unlawful disbursement process or anything will got me in here, I didn’t think about it at all. I thought, it’s easy, it’s okay, as long as they repayed it"

Code: Considering the behavior will not make any issues
REC33 checked_translated 171114

14.

"But I thought as long as they repay it regularly, that should be fine, safe. I didn’t think that when I went into politics, it would be used as a way to bring me down. At the beginning I was inspected, so when I became a campaigner, I would still work at my office"

Code: Considering the behavior will not make any issues
REC33 checked_translated 171114

15.

" saya pikir ga akan terjadi apa-apa."

Code: Considering the behavior will not make any issues
REC36

5.2.2.6. Considering could handle everything

1.

"Seakan-akan saya pake besok lusanya bisa saya ganti, kan seperti itu, kesombongannya di situ."

Code: Considering could handle everything
REC04

2.

"Bukan untuk direncanakan, saya dapat uang ini untuk tak ginikan. Waktu itu ga terpikir untuk anu, paling, soalnya sombonganya itu yang dianu yang ditonjolkan. Alaaah! Tak pake 1 juta 2 juta besok tak kembalikan, gitu kan?"

Code: Considering could handle everything
REC04

3.

"Berpikirnya belakang “Ahh! Tak gawe sewu, ngkok tak balekno” besoknya iya kalau ada rezeki, hehehe!"

Code: Considering could handle everything
REC04

4.

"Berarti ga mikir uang panas ini, dipake satu juta “Ahh! Besok dapat uang” lah ini loo tak ganti, padahal buat ganti ga ada"

Code: Considering could handle everything
REC04

5.

"Dan waktu itu saya masih punya kesombongan, artinya gini saya pasti bisa membayar. Dalam hati saya itu. Saya pasti bisa membayar sebanyak apapun yang saya tanggung, saya pasti bisa membayar. Ya dengan car awes gak karu-karuan tadi. "

Code: Considering could handle everything
REC27

6.

"Because I handled things up. If I didn't get an accident that time, it would be fine. Maybe because I got the accident, it's time to stop. Maybe it was safe because I did it all by myself"

Code: Considering could handle everything
REC32_translated_checked

7.

"I'm sure about it. Because I organized everything, the bank account, everything, was in my hands."

Code: Considering could handle everything
REC32_translated_checked

8.

"I was sure about it. I thought I won't get caught. I won't get caught because I handled everyting. That was basically my principle. I hold the control of the fundings. I was sure about it."

Code: Considering could handle everything
REC32_translated_checked

5.2.2.7. Didn't think about being sentenced/caught

1.

"(I have no bad prejudice about the legality. I didn't have any bad prejudice, I didn't think about being prosecuted, I wasn't thinking that far. I wasn't thinking about the legal issue."

Code: Didn't think about being sentenced/caught
REC02_translated_checked

2.

"Yes, certain amount was disbursed, then [they] cut some of it, you know. But I thought, I won't get in to jail, deal with the court, I have no clue."

Code: Didn't think about being sentenced/caught
REC11 checked_translated 171104

3.

" I didn't think that far, about being sentenced. I didn't, I don't understand law at all. I obeyed the suggestion from everyone, I know nothing else. I'm just a villager"

Code: Didn't think about being sentenced/caught
REC11 checked_translated 171104

4.

"No, I wasn't thinking that far. Well I was a totally blind about law, I didn't even feel that I [did it], so be it"

Code: Didn't think about being sentenced/caught
REC11 checked_translated 171104

5.

"You know. The issue about getting into prison, it didn't occur in my mind whatsoever."

Code: Didn't think about being sentenced/caught
REC11 checked_translated 171104

6.

"Ah, pling saya Cuma diberhentikan tidak sejauh ranah hukum karena banyak yang melakukan itu tapi mereka bagi-bagi dan saya nggak"

Code: Didn't think about being sentenced/caught
REC21

7.

"Yeah, the consideration was actually not caught. But the fact is, if I the accident didn't happen, maybe it won't be this far. Because of the accident, the only person who could disburse the fundings was me, I was the person who organized it. The chief, secretary, they only know the part where it got disbursed, but I did the fine-tuning, for this, and that. So I had to meticulously work on it."

Code: Didn't think about being sentenced/caught
REC32_translated_checked

8.

"I believe that it won't get noticed, if the accident did not happen."

Code: Didn't think about being sentenced/caught
REC32_translated_checked

9.

"I was sure about it. I thought I won't get caught. I won't get caught because I handled everyting."

Code: Didn't think about being sentenced/caught
REC32_translated_checked

10.

"So I didn't thought that the unlawful disbursement process or anything will got me in here, I didn't think about it at all"

Code: Didn't think about being sentenced/caught
REC33 checked_translated 171114

5.2.2.8. Considering could handle the risk

1.

Code: Considering could handle the risk
REC34

2.

Code: Considering could handle the risk
REC34

3.

Code: Considering could handle the risk
REC34

4.

Code: Considering could handle the risk
REC34

5.

Code: Considering could handle the risk
REC34

5.2.3. Common practice/Others also did

5.2.3.1. The leaders also did

1.
"Kalau atasnya sudah begitu kebawahnya itu ikut. Iya itu, inilah sistem"
Code: The leaders also did
REC01

2.
"karena dari atasnya memang begitu, itu yang paling berpengaruh. "
Code: The leaders also did
REC01

3.
"Nah kebetulan Dekan ku itu pernah juga dapat pekerjaan yang seperti ini. J"
Code: The leaders also did
REC16

4.
"Sekali lagi oknum yang dibawah melakukan itu karena di atasnya, istilahnya guru kencing berdiri, murid kencing berlari. Sepanjang guru kencing berdiri, jangan melarang murid kencing berlari. Tapi kalo diatas bener, dibawah sungkan. "
Code: The leaders also did
REC21

5.
"Eh kita nggak dikasih, direksi juga yang dapet.Ini kita yang marah, kita yang dilapangan. Cobalah kalo adil, gag mungkin kelakuan-kelakuan dibawah seperti ini salah karena contoh di atas nggak bener."
Code: The leaders also did
REC21

5.2.3.2. Others also did

1.
"Waktu itu saya membandingkan dengan Kabupaten-kabupaten lain, x, x, terkait dengan kebijakan seperti ini. Saya lihat mereka tidak ada masalah ya. "
Code: Others also did
REC05

2.
"Actually, almost everyone, other colleagues, do that as well. "
Code: Others also did
REC06_translated_checked

3.
"Sampean tak pinjemi bendera, tak gawe bendera CVmu. Tak kasih sekian. Terus kamu gak usah apa-apa tanda tangan sama stempel dengan tindakan nominalnya segitu. Belum ada yang ngatakan gak mau. Saya pun demikian. "
Code: Others also did
REC12

4.
"Semua lembaga pemikiran seperti itu terutama kampus kecil yang kekurangan mahasiswa. "

Code: Others also did
REC16

5.

"jelas. Ya menurut saya sudah cukup banyak ya, pertama saya ke Dekan terus tanya ke kampus-kampus lain yang sering berkegiatan seperti itu. Semua menyetujui, ""

Code: Others also did
REC16

6.

""Biasa pak H tu, lemlit-lemlit itu sudah biasa, banyak pekerjaan begitu, "

Code: Others also did
REC16

7.

"Ah, pling saya cuma diberhentikan tidak sejauh ranah hukum karena banyak yang melakukan itu tapi mereka bagi-bagi dan saya nggak. "

Code: Others also did
REC21

8.

Code: Others also did
REC23

9.

Code: Others also did
REC28

10.

Code: Others also did
REC34

11.

Code: Others also did
REC34

12.

Code: Others also did
REC34

13.

"Kebijakan dari direksi-direksi sebelumnya kan kek gitu. "

Code: Others also did
REC38

5.2.3.3. It was tradition (common practices)

1.

"No actually, because that's how it goes. Because I thought that was the tradition. If I had thought, well that's a small chance"

Code: It was tradition (common practices)
REC06_translated_checked

2.

"Nah kebetulan dekan ku itu pernah juga dapat pekerjaan yang seperti ini. Jadi sudah nggak asing, di kampus sudah nggak asing. Bagi kampus yang bonek-bonek itu, ambil aja. Pak dekan pernah pengalaman namun bukan x, x, x. Itu x itu mas,waduh.. uang dewan yang diacc DPR, eksekutif ya."

Code: It was tradition (common practices)
REC16

3.

"Masalah potong memotong itu biasa mas!!. Itu sejak zaman dahulu kala. Jadi potongan-potongan itu, kadang kecil kadang besar, jadi potongannya aja yang beda, ada 30%, 10% itu kan biasa, biasa jahat mereka gitu loh. Bukan biasa benar."

Code: It was tradition (common practices)
REC16

4.

"masalah potongan, kalo dana legal mereka sudah tahu, biasa mereka itu sudah biasa."

Code: It was tradition (common practices)
REC16

5.

"Biasa pak H tu, lemlit-lemlit itu sudah biasa, banyak pekerjaan begitu, yang nawarin sudah banyak. cuma ini potongannya terlalu besar". "

Code: It was tradition (common practices)
REC16

6.

" Itu terjadi sebelumnya. Kalau mau seperti itu semua, di balik Bank, di manapun, di PNS, itu yang terjadi. Nggak menutup mata kita. Hanya pilihan gubernur menyogok sana sini, sehingga yang lain…. Kalau dibilang "semua mencuri". Coba tanya kenapa saya yang ditangkap? Itu salah kan ? ya kita semua salah, ini mah sistimnya, "

Code: It was tradition (common practices)
REC19

7.

" Sistem nya emang sudah gitu, tdk ada sesuatu yang aneh,"

Code: It was tradition (common practices)
REC19

8.

"saya kira gag. Wajar lah sekali jalan dapat 250 ribu, itu kan x dibuat. Sistem itu sudah ada namun ketika di jalan bisa menjadi sistem yang baru yang tak tertulis,nikmati saja"

Code: It was tradition (common practices)
REC19

9.

"saya kira karena kasusnya 2011. Saya 2001 kembali. Kan 10 tahun, begitu sistem sebelumnya berjalan. Ada pajak yang kesini, ada pajak yang ksini. Yang kita harus laporkan ini full saya pakek semua. Gag ada pajak yang keluar. Jadi saya otomatis mengalir berjalan biasa"

Code: It was tradition (common practices)
REC19

10.

"ya karena sebelum-sebelumnya sistem berjalan seperti itu. Itu kebetulan ada masalah sama bosnya. Di bilang nasib, nggak. Di bilang kurang hati-hati juga nggak."

Code: It was tradition (common practices)
REC19

11.

"memang sistem sebelumnya sudah seperti itu. Di atas saya gag tahu, saya Cuma diyakinkan. Oleh .. . . Malah kita bayar DP duluan, padahal tahun depan cair (inden). Begitulah ngapain kita marah marah, mereka juga ada setoran lain. Jadi ada sedikit apatis, urusan dia ya urusan dia. Dia tak pernah ngurusin kita lagi, apalagi kita udah disini kan. "

Code: It was tradition (common practices)
REC19

12.

" tempat lain , grup juga. Kan sering kumpul. Sudah rahasia umum."

Code: It was tradition (common practices)
REC21

13.

"Disamping itu ada kata-kata orang masak bikin tumpeng, kita yang bikin tumpeng untuk dimakan begitu saja kita ga tau bagian, rata-rata seperti itu. Kita merencanakan, kita yang mendisign, kita yang menata, kita yang bikin tumpeng makanan ini kok enak dimakan hasilnya dikasih-kasih. Ini di mana-mana. "

Code: It was tradition (common practices)
REC36

14.

"sweetener itu pasti ke pihak ketiga yang memberikan. Dia kan punya fasilitas di penguruannya. Kedua terkait administrasi bank dan lainnya pasti ada. Pasti bank itu kita harus bayar di depan untuk dapet itu kayak seseiraang pengen dapet faasilitas 100% kan dia harus bayar adminnya 2% 1%, itu kan harus didepan. "

Code: It was tradition (common practices)
REC38

15.

"Kan itu kelaziman. Kalo di swasta itu marketing fee, kalo di swasta wajar pak, kalo misalnya setahu saya. "

Code: It was tradition (common practices)
REC38

16.

"Memberikan macem macem lah, tapi itu lumrah menurut kacamata saya loh ya, manusia kan butuh makan. Jadi ni menjadi cost kita."

Code: It was tradition (common practices)
REC38

5.2.4. Easy/simple way

1.

"The thing is we don’t want it to be complicated. We got the funding, we receive it, build it, that’s it."

Code: Easy/simple way
REC11 checked_translated 171104

2.
"then we [I] only do the signing, gave the photos, and got the money."
Code: Easy/simple way
REC11 checked_translated 171104

3.
"At that time Mr. x told us “for the areas that haven’t reached by the district, the province may give their funding” And I was, oh is it really that easy to process it through [him]. Oh here’s the chance. "
Code: Easy/simple way
REC11 checked_translated 171104

4.

Code: Easy/simple way
REC28

5.

Code: Easy/simple way
REC28

6.

Code: Easy/simple way
REC28

7.

Code: Easy/simple way
REC28

5.2.4.1. Low rate

1.
"dan yang kedua saya lihat bunganya ringan (0.5 %) . jadi petani kita juga tidak keberatan. Ya itu aja, daripada kita pinjem ke bank"
Code: Low rate
REC09

5.2.4.2. Less complicated

1.

Code: Less complicated
REC34

2.
"Jadi harusnya, kalo mau ambil resiko 0 saya ke minta APBD bangun pasar tapi kan prosesnya 1 tahun atau 1,5 tahun. "
Code: Less complicated
REC38

3.

"pertama karena kecepatan kerja sedangkan di APBD itu anggarannya bisa 2 tahun, target kita kan sebagai di Direksi 4 Tahun."

Code: Less complicated
REC38

4.

"Trus efisiensinya jelas, ke swastsa kita cuma laporan, nggak ribet karena pakek dana pihak ketiga. "

Code: Less complicated
REC38

5.

"salah satunya kecepatan kerja kita mendapatkan funding tanpa resiko, report gampang, dan yang terakhir tidak pakek dana pemerintah yang menyebabkan reportnya menjadi sulit. Dana APBD itu sulit. Orang diperiksa, audit, sulit pak. Lebih sulit 2x dari swasta. Saya terakhir pernah dapet CSR dari x nilainya 3,5M, dormitory di x, saya dari tongtong x tahun 2004. Udah saya bangun dormitory, trus dilihat, bener yaudah pulang. Pertanggungjawabanya lebih simple."

Code: Less complicated
REC38

5.2.4.3. Doable

1.

"Dan itu saya berhasil, berhasil mendapat dana kembali. Karena ga semua bisa. Mungkin terlalu pinter kayaknya.
(I succeed to get the fundings back. Not all of them can. Cleverness, perhaps.)
P: Dan bisa dilakukan?
(And it was doable?)
R: Bisa dilakukan, iya itu dapat lagi kan. Dapat dari pemikiran saya sendiri aja.
(It was doable, that was why [we] got it. It just came to my mind.)"

Code: Doable
REC32_translated_checked

5.2.4.4. Didn't do anything, got the money

1.

"Fee itu berapa persen dikasih? Saya wong gak melok kerja ya, gak lapo-lapo dikasih 50 juta ya siapa yang gak mau? "

Code: Didn't do anything, got the money
REC12

5.2.4.5. Fast way

1.

"Kita pengen cepet. "

Code: Fast way
REC19

2.

"pertama karena kecepatan kerja sedangkan di APBD itu anggarannya bisa 2 tahun, target kita kan sebagai di Direksi 4 Tahun."

Code: Fast way
REC38

5.2.5. Change

1.
"yang pertama, memang kesempatan"
Code: Change
REC01

2.
"Kesempatan, Yang pertama, kesempatan, kesempatan ini"
Code: Change
REC01

3.
" kesempatan"
Code: Change
REC01

4.
"Saya pikir ini rejeki aja. Saya pikir ini rejeki aja, makelaran, dapat makelaran gitu lo."
Code: Change
REC03

5.
"Bukan yang terakhir kok kesempatan yang mendukung."
Code: Change
REC19

5.2.5.1. There was a buyer

1.

Code: There was a buyer
REC37

2.

Code: There was a buyer
REC37

3.

Code: There was a buyer
REC37

6. Behavior

6.1. Conspiracy victim

1.
"Yang keduanya lagi, sekan-akan semua yang dibuat BAP polisi betul –betul terjadi. Ini terbukti karena apa di saat petikan putusan jadi, itu persis sekali dengan BAP yang dibuat polisi, ndak ada perubahan sama sekali. Jadi by setting dari awal supaya saya dimasukkan, dikriminalisasi seperti ini"

Code: Conspiracy victim
REC09

2.

Code: Conspiracy victim
REC26

3.

Code: Conspiracy victim
REC26

4.

Code: Conspiracy victim
REC30A

5.

Code: Conspiracy victim
REC30B

6.

Code: Conspiracy victim
REC37

6.1.1. Being appointed as project committee

1.

Code: Being appointed as project committee
REC23

6.1.2. Not involved in the project committee

1.

Code: Not involved in the project committee
REC23

2.

Code: Not involved in the project committee
REC29A

6.1.3. Selling the land to the state company

1.

Code: Selling the land to the state company
REC37

6.1.4. Giving the loan

1.

Code: Giving the loan
REC17

2.

Code: Giving the loan
REC17

3.

Code: Giving the loan
REC20

6.1.5. Borrowing money to the state company

1.

"Saya ngelola, 2,3 ini dari PT.x, diberikan lagi ke saya kan dari PT. xy. 2,3 diberikan ke saya berupa benih padi, pupuk dan biaya garap sawah. Itu yang dipinjamkan ke saya, ini pinjaman. Pada kronologis ini petani banyak yang gagal karena benih dari PT.x ini tidak bagus dan diawal sudah kami tolak, tidak bagus. Tpi PT.x bilang "jalankan aja". Begitu gagal beneran, saya nuntut PT.x, malah saya suruh ganti"

Code: Borrowing money to the state company
REC09

6.1.6. Assisting farmer community for productivity

1.

Code: Assisting farmer community for productivity
REC26

6.1.7. Buying the land for the sugar factory

1.

Code: Buying the land for the sugar factory

REC26

6.1.8. Being considered as the state company staff

1.

Code: Being considered as the state company staff
REC37

6.2. Wrong policy/administration

6.2.1. Signing contract for exploring natural resource for private co

1.

Code: Signing contract for exploring natural resource for private co
REC35

2.

Code: Signing contract for exploring natural resource for private co
REC35

6.2.2. Building village market in the disputed land

1.

Code: Building village market in the disputed land
REC30A

6.2.3. Giving the money for a project without a proper receipt

1.

"Jadi begini, memang ada kekeliruan dari saya, kesalahan saya. Kesalahan saya adalah memberikan uang tanpa tanda terima, tapi uang ini sudah digunakan, uang Negara ini sudah digunakan oleh panitia. Kemudian digunakan, ada semuanya barangnya, distributornya iya ada, suplaiernya iya ada, barangnya iya ada, laporan pun sudah dibuat."
Code: Giving the money for a project without a proper receipt
REC01

2.

"Kesalahan saya hanya tidak pakai tanda terima memberikan uang."
Code: Giving the money for a project without a proper receipt
REC01

6.2.4. Spending money that was not included in the budget

1.

Code: Spending money that was not included in the budget
REC18

2.

"Akhirnya kita direksi dianggap menyalahi prosedur, mengeluarkan uang tidak sesuai peruntukan, memang nggak ada budgetnya mengeluarkan uang tanpa peruntukan. Tapi waktu kita menganggap uang keluar bukan sebagai cost tapi uang muka. Jadi misalnya ada budget di perusahaan, oowh ini bukan peruntukan, misalnya uang untuk nasabah, kita pakek dulu deh sebagai uang muka."

Code: Spending money that was not included in the budget
REC38

6.2.5. Didn't realize the project grant

1.

Code: Didn't realize the project grant
REC13

2.

Code: Didn't realize the project grant
REC13

3.

Code: Didn't realize the project grant
REC13

6.2.6. Land acquisition without appraisal process

1.

"Saya dinyatakan salah karena tidak pakek “appraisal” (penaksir) di satu sisi kita sudah meminta bantuan ke bagian pajak karena waktu itu pihak pajak mengatakan tidak mau menjadikan apraisal mundur terus kita melakukan musyawarah mufakat"

Code: Land acquisition without appraisal process
REC24

2.

"Jadi yang dianggap kesalahan ibu dimana ya?
P: tidak memakai appraisal, menyatakan bisa menimbulkan kerugian negara"

Code: Land acquisition without appraisal process
REC24

3.

" Jadi waktu itu memang dianggap kelalaian dimana bu?
P: Tidak ada appraisal
R: Appraisal maksudnya gimana bu?
P: Tim penafsir atau Lembaga penafsir, kofido kalo sekarang"

Code: Land acquisition without appraisal process
REC24

4.

" sehingga disepakati harga sekian?
P: iya,
R: lewat musyawarah mufakat?
P: iya dengan pemilik tanah langsung, tidak diwakili"

Code: Land acquisition without appraisal process

REC24

5.

"Tidak, karena kita sudah kirim surat ke provinsi, ke pajak tuk ditunjuk sebagai appraisal/ tim penaksir. Kita minta petunjuk ke provinsi, sekda juga gag ada. Satu sisi surat dari PU dan provinsi pusat berdatangan akhirnya kita melibatkan MUSPIDA salah satunya kan kekhawatiran pasti ada, tapi karena sudah dengan musyawarah mufakat, harga juga disetujui. "

Code: Land acquisition without appraisal process
REC24

6.

"Karena tidak menggunakan appraisal saya dianggap bisa merugikan negara. "

Code: Land acquisition without appraisal process
REC24

7.

"Dan itu tetap dinyatakan menyalahi ibu?
P: Iya, dari situ muncul tetep tidak menggunakan appraisal"

Code: Land acquisition without appraisal process
REC24

6.2.7. Investing money to the business outside organization's vision

1.

Code: Investing money to the business outside organization's vision
REC14

6.2.8. Borrowing money to the bank with wrong procedure

1.

Code: Borrowing money to the bank with wrong procedure
REC28

6.2.9. Giving the credit without proper management

1.

Code: Giving the credit without proper management
REC34

2.

Code: Giving the credit without proper management
REC34

3.

Code: Giving the credit without proper management
REC34

4.

Code: Giving the credit without proper management
REC34

6.2.10. Signing the receipt for the old project

1.

Code: Signing the receipt for the old project
REC18

6.3. Favouritism

6.3.1. Harming the organization and giving the benefit to other party

1.

Code: Harming the organization and giving the benefit to other party
REC22

6.3.2. Giving the loan from organizational budget to the football club

1.

Code: Giving the loan from organizational budget to the football club
REC22

2.

Code: Giving the loan from organizational budget to the football club
REC22

6.3.3. Giving the loan to the ineligible people

1.
"But here's where I was wrong, I knew I was wrong, but I thought it wasn't violating the PTU, the instruction manual, [the places] where it should be developed, I dared to do it, so I took the liberty. The money, as long as its... So [the budget] won't be freezed [idle]. I rolled it, but it made me got in to this corruption case, I rolled [gave] it to the people who was not eligible, to the sub-district head, local police head, village head. Yet it is forbidden in the instruction manual. But I thought it was better than [if the grant was] idle, rather than freezed, I was, let's say, when those people came to me, such as the sub-district head, local police head, or village head, I made promises. I promised, the point is [for them] not to be late for the repayment. And everything went well. Even in 2007, I got an award from the Ministry of x, because it was considered as very quick"
Code: Giving the loan to the ineligible people
REC33 checked_translated 171114

2.

" It also went to the [ineligible] people, say, the district head, village head, or to the local police head, but [the receiver name was] disguised. But they still the one who received the money"

Code: Giving the loan to the ineligible people
REC33 checked_translated 171114

3.

"When I allocated it to the ineligibles, I realized they were the wrong ones, but why, because the lower class was no longer need it. I mean, all of them were covered, I only allocated the money [to the ineligible ones] when the money was idle. So I disbursed it when there were freezed [unused] funds."

Code: Giving the loan to the ineligible people
REC33 checked_translated 171114

4.

Code: Giving the loan to the ineligible people
REC34

5.

Code: Giving the loan to the ineligible people
REC34

6.3.4. Giving the loan from organizational budget to small enterprise

1.

Code: Giving the loan from organizational budget to small enterprise
REC14

6.4. Assisting/involved

1.

"Karena saya dianggap ikut membantu di dalam penyelewengan itu."

Code: Assisting/involved
REC07

2.

"On where the money go, and I was only ordered to make a fictious report that the money has been spent on such and such. They asked the same thing in 2013, starting from that point... Well the signature was mine, why didn't I get any and I didn't feel that they returned the money and none of it was channeled to the government"

Code: Assisting/involved
REC10_translated_checked

3.

"Dan saya ikut mendukung, otomatis kan mendukung itu. Jadi, saya ya memang salah cuma vonisnya gak seperti itu tidak. Pikir saya kan kalau seperti itu paling tidak satu tok saya mengembalikan semua 50 juta dan denda 50 juta."

Code: Assisting/involved
REC12

4.

"Dianggap ikut bersama-sama dan membantu kalau gak ada CV saya."

Code: Assisting/involved
REC12

6.4.1. Assisting in money movement process

6.4.1.1. Being asked to issue some money for unclear expenses

1.

" Fraud was indeed happened there. In 2011, I was in regional revenue departement. I had my suspicion when I was told to sign for issueing some money. I did have the authority in district S, and was entrusted for that 2B [billion], for a year. In 2011 the regional head asked me to issue some money, more or less 800 million, sir. And I was like, "Why is it not returned?")
P: Uang gak kembali ini ke mana?
(The money, not returned, what do you mean?)
R: Uang itu ke mana dan saya cuma disuruh laporan fiktif kalau sudah dibelanjakan ini ini ini. Diulang lagi 2013, akhirnya dari situ, wong namanya orang tanda tangan itu saya ya, kok saya gak dapat apa-apa dan saya merasa uang itu tidak dikembalikan sama mereka dan gak ada yang disalurkan ke pemerintahan.
(On where the money go, and I was only ordered to make a fictious report that the money has been spent on such and such. They asked the same thing in 2013, starting from that point... Well the signature was mine, why didn't I get any and I didn't feel that they returned the money and none of it was channeled to the government.)"

Code: Being asked to issue some money for unclear expenses
REC10_translated_checked

2.

Code: Being asked to issue some money for unclear expenses
REC29A

6.4.1.2. Taking and giving the money from corruption

1.

"Begitu proposal diacc sama gubernur uangnya kan masuk ke rekening lembaga sana, saya bagian mengambil. Nah, ini saya terkena, ada tanda bukti. Uang itu diserahin ke Pak X."

Code: Taking and giving the money from corruption
REC16

2.

"Karena tanda terima uang dari lembaga lewat saya, saya yang tanda tangan, diserahin ke Pak X. Nah Pak X ini tidak ada tanda bukti."

Code: Taking and giving the money from corruption
REC16

3.

"Saya ini tanda tangan mengambil dana untuk diserahkan kepada yang punya pekerjaan. Nah kenapa dia nggak mau ambil sendiri, kan ndak kenal dengan lembaga-lembaga LPM, Lemlit tadi itu. "

Code: Taking and giving the money from corruption
REC16

4.

"Yang perintah kan saya, yang pesen Pak E ke sini. Pak E dari Pak B, Pak B dari orang dewan mungkin ya. Pak B ke Pak E, Pak E ke saya. Saya ke lembaga. Jadi semua lembaga mengatakan yang nyuruh Pak H (subyek)."

Code: Taking and giving the money from corruption
REC16

5.

"Dasar pak E ini, saya capek bawa-bawa uang dan takut bawa uang segitu banyak. Kadang diantar ke kampusnya kadang diantar kerumahnya, kadang diambil di rumah saya. "

Code: Taking and giving the money from corruption
REC16

6.4.1.3. Helping in process of issuing the money

1.

Code: Helping in process of issuing the money
REC23

2.

Code: Helping in process of issuing the money
REC29B

3.

Code: Helping in process of issuing the money
REC29B

6.4.1.4. Helping in the taking money

1.

"60% dilaksanakan, 40% yang diambil."

Code: Helping in the taking money
REC19

6.4.1.5. Being asked to create a new bank account

1.

Code: Being asked to create a new bank account
REC29A

6.4.2. Making the false document

1.

"Jadi, sama kepala kalau disuruh ngetik ini, ya saya ketik. Tugas mengetik itulah yang dianggap saya memalsukan dokumen dst. "

Code: Making the false document
REC15

2.

"Cuma sebatas ngetik itu tadi?
R: Iya. Ngetik dokumen dianggap saya merekayasa dokumen itu. "

Code: Making the false document
REC15

6.4.3. Helping in land acquisition process

1.

"Ohh iya Pak saya bantu ini, ohh iya Pak saya bantu, ohh iya Pak saya nego, ohh iya Pak saya ngantar orang bayar, ohh iya Pak saya ngantar uang bayar”"

Code: Helping in land acquisition process
REC03

2.

"Eeeeem, jadi saya di situ didakwakan ikut membatu aja. Ikut membantu memperkaya orang lain. Jadi pasal yang didakwakan ke saya itu ikut membantu"

Code: Helping in land acquisition process
REC03

3.

"Saya disitu bagian apa? Saya yang banyak itu bagian surat-surat, jadi di legalitas itu sama perizinak saya yang banyak di situ, jadi pajak, surat sama pajak biasanya saya dilibatkan di situ. Jadi juga yang di kampus U ini saya banyak untuk surat dan pajak. Jadi saya membantu bikin surat-suratnya."

Code: Helping in land acquisition process
REC03

4.

"Jadi begitu dimintai bantuan, iya sudah bergerak. Jadi mulai pengumpulan data, data masyarakat itu iya, sampai nyari alamatnya, alamat orang-orang itu. Jadi kita bagi-bagi pekerjaan dengan temen-temen yang lain.
R : Ohh gitu?
P : Iya, jadi ada yang ke desa ini, ada yang ke daerah ini nemui orangnya terus minta fotocopynya setelah itu kita serahkan pada salah satu orang kepercayaan panitia kampus U itu. Terus kita nunggu kabarnya, jadi seperti itu. "

Code: Helping in land acquisition process
REC03

5.

"Pak, Pak Carik tulung sampean terno, mbawa kendaraan sampean”. Iya sudah nganter ke Bank, dibayar di Bank. Dia bawa uang kes pulangnya, ngawal sampai rumahnya terus diamplopi, iya seperti itu. Jadi yang banyak seperti itu. terus ada juga yang “Pak Carik ini mau saya jual cuman surat saya ini petok D” Sedangkan kampus U nerimanya yang jual suratnya itu berbentuk akte.
R : Hak milik?
P : He’em, Akte. Iya wes saya bikin kan surat, saya bikinkan surat. “ini pak tanda terimaksih karena suratnya sudah selesai cepat” seperti itu."

Code: Helping in land acquisition process
REC03

6.4.4. Lending the company for the corrupt project

1.

"Jadi saya punya CV dan itu berjalan ya, maksudnya eeeeh…aktif. Saya waktu itu dipinjam untuk dipakai temennya saudara saya, untuk dipakai temennya di kota S yang seakan-akan tadi untuk proyek pengadaan kain batik kabupaten N. Eeeeh."

Code: Lending the company for the corrupt project
REC12

2.

" Bendera saya dipinjam, jadi saya punya perusahaan bergerak di bidang jasa konstruksi. Bendera saya dipinjam oleh seorang kawan, saya ga biasanya seperti itu. Terus dia datang menjelaskan pengerjaan proyek yang sudah dikondisikan oleh pejabatnya, oleh yang punya kekuasakan tersebut. "

Code: Lending the company for the corrupt project
REC36

6.4.5. Managing the event

1.

"Nah, kalau Pak E gak mampu menjalankan pelatihan minta tolong saya. Saya buat tim kecil-kecilan 3 orang, 4 orang, gitu datang ke daerah dan pelatihan. Lha yang berpelatihan pematerinya ya kampus-kampus terkait itu. Undang ke sana, kamu punya hajat. Itu yang bicara sana, saya hanya ngatur aja ngatur waktu, itu gitu."

Code: Managing the event
REC16

6.4.6. Finding institution as partner in corruption process

1.

"Jadi dalam kasus saya, saya hanyalah seseorang yang diminta bantuan oleh teman yang kebetulan teman itu adalah yang mempunyai jabatan di suatu institusi, perguruan tinggi swasta yang kemudian meminta tolong kepada saya apakah saya mempunyai teman yang mempunyai perusahaan dengan spesifikasi tertentuS. saat itu saya mengatakan kalo saya sendiri nggak ada pak. "Tapi coba saya akan tanyakan pada temen saya", seperti itu akhirnya berkembang terus memang ada teman saya mempunyai beberapa rekan gitu dengan spesifikasi yang teman saya katakanlah "A" yang beliau A ini minta. "

Code: Finding institution as partner in corruption process
REC07

2.

"Seharusnya seperti itu, bukan saya. Nah karena saya dianggap menyediakan, memperkenalkan tender yang tidak sebenarnya, itu dianggap bukti
R: Cuma dianggap sebagai memperkenalkan?
P: iya, tapi memang begini. Pihak perguruan tinggi yang tahu saya yang tahu perusahaan. Jadi saya sebagai jembatannya, istilah kasarnya saya jadi broker nya. Jadi, perguruan tinggi ini hanya tahu saya, tidak tahu perusahaaan. Ya tahu namanya tidak tahu direkturnya siapa."

Code: Finding institution as partner in corruption process
REC07

3.

"Dia minta tolong saya mencarikan lembaga penyalur dana ini. Saya kan dosen, saya kenal banyak tentang PTS di seluruh provinsi. Tinggal kontak, datangi, datang, siap jalan dengan pekerjaan ini. Saya kan gak pernah jahat, jadi orang percaya aja. Begitu ngomong percaya. Akhirnya dapat 4 kampus, di kota A 2 kampus, B, terus universitas U di kota B."

Code: Finding institution as partner in corruption process
REC16

4.

"Saya bantu dia aslinya itu, malah saya yang bekerja. Saya bantu dia mencarikan lembaga, titik. "

Code: Finding institution as partner in corruption process
REC16

6.4.7. Falsifying financial report

1.

"Where the money go, and I was only ordered to make a fictious report that the money has been spent on such and such. They asked the same thing in 2013, starting from that point... Well the signature was mine, why didn't I get any and I didn't feel that they returned the money and none of it was channeled to the government"

Code: Falsifying financial report
REC10_translated_checked

2.

"Di ini, jadi pemakaian, ya jelaslah kalau misal kita ambil 100, pertanggungjawaban saya cuma 50, 50 nya lagi saya karangkan."

Code: Falsifying financial report
REC19

6.4.8. Signing the document

1.

" That I'm the victim. Because I signed documents that I don't even understand. It supposed to be for somebody else, but it was given to me, which I don't understand anything about it. In the document, I was representing one of the sellers. "

Code: Signing the document
REC02_translated_checked

2.

"The violation was about signing the document, which represented 70 people"

Code: Signing the document
REC02_translated_checked

3.

"I did the signing"

Code: Signing the document
REC02_translated_checked

4.

"Then I was given the document with my name on it. I signed it afterwards."

Code: Signing the document
REC02_translated_checked

5.

" Oh! It appears that the xxx document, the list of people who sold their land, was signed by me."

Code: Signing the document
REC02_translated_checked

6.

" So I signed the document for the disbursement, to pay those sellers. At that time, I just comply with what was ordered."

Code: Signing the document
REC02_translated_checked

7.

"Yes, the signature, well actually I wasn't wrong. I was told to sign it."

Code: Signing the document
REC02_translated_checked

8.

"Then we [I] only do the signing, gave the photos, and got the money. "

Code: Signing the document
REC11 checked_translated 171104

9.

" Jadi intinya saya hanya stempel dan tanda tangan. Eeh… untuk yang mengerjakan itu orang kota situ yang punya proyek. Saya gak ngerti gak tahu masalahnya apa eeeeh… apa yang dikerjakan yang jelas untuk pengadaan kain batik. Betul bidangnya itu ada. Memang dan saya sendiri sebenarnya sudah, CV itu eeeh… udah maksudnya aktif itu cuma saya…."

Code: Signing the document
REC12

10.

Code: Signing the document
REC23

11.

Code: Signing the document
REC29A

12.

"Kontrak, habis itu lepas. Tanda tangan kontrak itu dilampiri dengan surat kuasa tadi. Berkasnya menunjukan sesuatu."

Code: Signing the document
REC36

6.4.9. Getting money from the failed credit

1.

Code: Getting money from the failed credit
REC31

2.

Code: Getting money from the failed credit
REC31

6.4.10. Helping in the finding the new creditors

1.

Code: Helping in the finding the new creditors
REC28

2.

Code: Helping in the finding the new creditors
REC28

6.5. Bribe

1.
"At least 600 [million]. With giving up 50-70 [million] we got the amount of 600-700 [million], well, that's good enough. In the end, there was the officer who sent it [bribe] in, I was not involved, as long as I gave my approval. It's like the ancient proverb, peri-peri koloso, to be a little bit in the dangerous side.)"
Code: Bribe
REC06_translated_checked

2.
" I proposed it since I was appointed back to the school, I've been working on that land (acquisition) from 2010. It was failed in 2010, 2011, because I did not agree on the additional "fee". When I said, "OK", then there it goes, straight away in 2013.)"
Code: Bribe
REC06_translated_checked

6.5.1. Giving bribe

1.
"It was because of the cooperation from the other colleagues, I was only coordinating them. The thing was, in the circle of the Ministry of X, if we want to get some projects going on, we have to be bold, giving some to [bribe] people in the head office. So, the every budget should have their approval. I always coordinate with the Committee. Let's say "Sir, what if we are willing to pay 50-70 million?" "
Code: Giving bribe
REC06_translated_checked

2.
"Yup. That was why I took this way. If there's an issue, then I think that's the risk.)"
Code: Giving bribe
REC06_translated_checked

3.
"Akhinya kita mencari funding dari pihak ketiga. Hibah lah istilahnya, nah kita urus. Bapak tahu kan istilahnya kan bapak kalo mau mincing harus ada kailnya, ada umpannya. Kalo bahasa saya di swasta itu, jadi umpan itu harus kita beli kan dengan kemampuan kita. Makanya uang itu keluar untuk sweetener lah istiahnya. "
Code: Giving bribe
REC38

4.
"Yang kedua mengenai pajak. Jadi pajak dulu perusahaan pasar itu ada yang belum bayar pajak, sampek sekarangpun begitu. Saya mengajukan yang namanya keberatan pajak, tapi tetep pak yang namanya dalam proses negosiasi ada yang namanya sweetener supaya, yang kasus pertama uang hibahnya keluar, yang kedua pajaknya diterima."
Code: Giving bribe

REC38

6.6. Manipulation of information

6.6.1. Mark-up

1.

"Mau ga mau harus dimark-up harga itu"

Code: Mark-up
REC06_translated_checked

6.6.2. Making the false document

1.

"Jadi, sama kepala kalau disuruh ngetik ini, ya saya ketik. Tugas mengetik itulah yang dianggap saya memalsukan dokumen dst. "

Code: Making the false document
REC15

6.6.3. Falsifying creditor identity

1.

"It also went to the [ineligible] people, say, the district head, village head, or to the local police head, but [the receiver name was] disguised. But they still the one who received the money.)"

Code: Falsifying creditor identity
REC33 checked_translated 171114

2.

"For the ledger, I made it in behalf of a name, using a false name"

Code: Falsifying creditor identity
REC33 checked_translated 171114

3.

Code: Falsifying creditor identity
REC34

6.6.4. Manipulating the data to get the loan

1.

"Jadi akhirnya mereka minta tolong ke saya melalui kantor saya. Nah pemotongan gajinya harus dari kantor saya. Berhubung di luar kantor saya jadi tidak bisa terpotong. Jadi, mereka membayar sendiri leawat saya. Ternyata setelah pencairan dana, saya waktu itu bekerja sama dengan bendahara saya, bendahara pengeluaran, dibuatkan SK palsu terus ditandatangani juga pimpinan saya yang memalsukan tanda tangan. Kebetulan saya bertiga kerja sama satu kantor."

Code: Manipulating the data to get the loan
REC25

2.

"Nah tanggung jawab saya itu menempuh cara yang salah. Saya mencari nasabah lain, temen saya dibuatkan SK palsu oleh temen saya. Akhirnya, jadi non pns. Orang tersebut bukan PNS tapi kami jadikan seolah olah PNS. Jadi berputar disitu trus, akhirnya menumpuk"

Code: Manipulating the data to get the loan
REC25

3.

"Jadi saya minta tolong teman di kecamatan lain untuk meminjamkan uang di bank. Atas nama saya seakan-akan saya adalah pegawai di kecamatan lain tersebut. Saya berdinas di kecamatan K minta teman saya di kecamatan KK."

Code: Manipulating the data to get the loan
REC27

4.

"Akhirnya saya memalsukan data.. memalsukan data, kebetulan di Bank X. Memalsukan data untuk mendapatkan pinjaman lebih atas nama kecamatan KK ini tadi. Eee.. saya memakai orang-orang palsu yang sebenarnya bukan pegawai negeri, tetapi saya meminta mereka untuk meminjam, seakan-akan mereka pegawai negeri. Saya membuatkan mereka SK palsu dan seluruh administrasi palsu, dan hal itu ternyata berhasil. "

Code: Manipulating the data to get the loan
REC27

5.

Code: Manipulating the data to get the loan
REC34

6.6.5. Building the road was not according to the grant agreement

1.

" Well actually what was arranged in xxxx was 2.5 meters [road width], but I built 2 meters. But that 50 [million]? The work force was even the villagers themselves"

Code: Building the road was not according to the grant agreement
REC11 checked_translated 171104

6.6.6. Falsifying the financial report

1.

"Where the money go, and I was only ordered to make a fictious report that the money has been spent on such and such. They asked the same thing in 2013, starting from that point... Well the signature was mine, why didn't I get any and I didn't feel that they returned the money and none of it was channeled to the government"

Code: Falsifying the financial report
REC10_translated_checked

2.

"Di ini, jadi pemakaian, ya jelaslah kalau misal kita ambil 100, pertanggungjawaban saya cuma 50, 50 nya lagi saya karangkan."

Code: Falsifying the financial report
REC19

3.

"a, APBD. Daerah ya. 25 diambil sana gimana pertanggungjawabkannya. Saya kan harus mempertanggungjawabkan 100 kan. Ketika diperiksa kok cuma 50, gimana? Lah memang 50 kenyataannya saya terima. Ya saya harus markup. "

Code: Falsifying the financial report
REC19

4.

"Sering, dipotongin juga gitu. Di balai bank jg, terima 250. Setengahnya malah. Dan harus buat laporan full, padahal enggak. "

Code: Falsifying the financial report
REC19

5.

"Yeah, from 2013. As long as in 2012 we don't have any outstanding payment. And me, as the treasurer, should be smart, so that we got the funding again. So we... I, with an approval, I was not, I mean I was not alone. So I have to go with the chief and the FK, FK is like the supervisor. District Facilitator [FK]. There were two, tekchnisiant empowerment. We have to sign it. I ask for the signature, I forged, as long as [we] get the funding again.)
P: Tanda tangan dari?
(Signature of?)
R: Ketua sama FK. Itu juga ada persetujuan dari kecamatan trus kabupaten, itu.
(The chief and the district facilitator. There were also approval frorm the district and the regency.)
P: Yang difiktifkan itu tandatangan?
(So was it the signature that was faked?)
R: Laporan
(The report)
P: Laporanya?
(The report?)
R: Laporanya aja, kita kan mendapatkan dana tanpa dari atas melihat fisiknya. Jadi kita cuman dipercaya cuman lihat datanya aja. Datanya kita nol, ga ada tunggakan, dapat dana kembali, itu.
(Only the report, we've got the funding without looking at the physical evidence. So we were trusted by only looking at the data. In the report, the [credit] balance was zero, there was no outstanding, [so we] got the funding back again. That's it.)"

Code: Falsifying the financial report
REC32_translated_checked

6.

"Yes, the reports, I faked the reports. There was no money during the fund disbursement, no money, it was from the district, when we disburse it.)"

Code: Falsifying the financial report
REC32_translated_checked

7.

"(So what lated cosidered as the crime was the faking the report?)
R: Memalsukan laporan, terus tidak bisa dikembalikan karena itu macet.
(Faking the report, then it was not refundable because of its bad cradit.)"

Code: Falsifying the financial report
REC32_translated_checked

6.6.7. Executed the event that was not according to the budget plan

1.

"Jadi saya ini kan pelaksana teknis. Pada waktu melakukan kegiatan sosialisasi itu memang ada dana yang tidak sesuai dengan kerangka anggaran. Dana yang dianggarkan sekian tapi yang dikeluarkan sekian. Itu kan tidak sesuai. Tapi itu semua sudah ada nota kebijakan pimpinan untuk dilaksanakan meskipun tidak sesuai. "

Code: Executed the event that was not according to the budget plan
REC05

6.7. Embezzlement

6.7.1. Borrowing money from the project budget

1.

Code: Borrowing money from the project budget
REC34

2.

Code: Borrowing money from the project budget
REC34

6.7.2. Taking the money

1.

" Iyaaa, mengambil bukan haknya kan. Mengambil bukan haknya. Itu lah dosa diri. Orang masuk kesini karena dosanya. Tapi belum tentu kasusnya itu yang dosanya. Kasus itu hanya untuk jalan masuk ke sini. "

Code: Taking the money
REC01

2.

" Kalau itu, tepatnya apa Pak yang pernah dilakukan?
P : Mengambil yang bukan haknya"

Code: Taking the money
REC01

3.

"Saya pernah melakukan mengambil yang bukan hak saya tapi saya masuk buakan karena itu. Itulah pokoknya, yang saya perbuat itu ndak masukan saya ke sini tetapi yang tidak saya perbuat yang memasukan saya ke sini. "

Code: Taking the money
REC01

4.

"Saya akui, seperti tadi. Saya akui uang itu saya pegang memang, habis memang iya, kan gitu."

Code: Taking the money
REC04

5.

"Uangnya sekarang di mana sisanya?" "Ada saya bawa pak" "Sekarang?" "Ada dirumah" "Ambil aja", iya kan, ambil aja. Orangnya ambil saya bawa. Dikasihkan ke saya waktu itu tak pegang, lah ini kesalahannya itu. Seharusnya kan memerintahkan aja, kenapa saya harus pegang. Otomatis besoknya saya dikasih kwitansi untuk membawa sisanya itu yang 20 juta itu, lah itu loo, iya kan."

Code: Taking the money
REC04

6.

"Waduh, saya ga, waktu itu kan namanya orang ada aja kan. Ga langsung habis uang segitu, ga langsung habis, kan. Ga tahu saya pegang itu sehari keluar satu juta atau dua juta kan ga terasa."

Code: Taking the money
REC04

7.

"Kita ga mungkir iya, seperti saya 48 juta ngembil di Bank X, ini jatahnya sendiri-sendiri sudah ada, yang pengajuan proposal terus lainya walaupun ndak tertulis ini, kan, hanya Tuhan yang tahu. Memberi memperlancar proposal ini kan sampean bantu saya untuk tak ajukan proposal. “Sudah sampean tak batu proposalnya, tak ajukan nanti” apa rasa trimakasih saya ke sampean hanya trimakasih tok? Kan ga mungkin? “Ini mas uangnya sudah cair, sampean tak kasih ini iya?” kan ndak tertulis. Tapi nominal pertanggungjawaban SPJnya ini tetap 48. Sebelum dilaksanakan proyek sudah 48 sudah hampir 40 yang 8 juta sudah diberikan, SPJnya tetap 48."

Code: Taking the money
REC04

8.

" saya bisa memberikan biaya operasional untuk menambah gaji staf itu juga ke bagian keuangan trus kedua saya ambil tagihan itu dan saya salurkan ke orang-orang tadi yang kelaparan."

Code: Taking the money
REC21

9.

"Gag sekaligus pak, saya ambil kalo ada kesempatan sekian karena 3 tahun saya menjabat jadi manager. Sebelumnya saya asisten manager.
R: kalo nominal yang dituduhkan berapa?
P: 1 M, yang benar-benar saya lakukan 400 juta dan saya akui seinget saya . Cuma saya gag pernah nyatet. Itu kan dari klien yang belum ditagih, dituduhkan bayar ke saya."

Code: Taking the money
REC21

6.7.3. Getting/obtaining money from the project

1.

" dan seharusnya saya ndak boleh menerima pemberian itu, ndak boleh. Maksimal pemberian yang diberikan kepada itu harusnya paling banyak 2 juta, pekerjaan satu tahun itu. Tapi pada saat itu tidak saya jelaskan saya diberi siapa. Total semua itu saya diberi masyarakat juga, masyarakat itu banyak, puluhan orang, nah itu saya dikasih begitu juga dari kampus X saya kumpulkan jadi satu ada sekitar 76 juta itu. Lah sekarang, pada saat itu masyarakatnya sekitar 74 orang. Lah kalau per orang ngasih saya karena saya membantu surat-suratnya, satu juta jadi 74 juta. Di kasih dari kampus, memang dari kampus itu saya dikasih sekitar 14 kalau ga salah, ohh 17 juta."

Code: Getting/obtaining money from the project
REC03

2.

"“So, this is for the construction, for your expenses, well, you went to a city, and for the community group and its operational expenses, your effort, for the sub-district head, and I take 1000ish [around 1 million]. 47 [million] left. "

Code: Getting/obtaining money from the project
REC11 checked_translated 171104

3.

" Every time we got the disbursement, I got 50, and I came to a certain neighborhood. Here's the grant, I set aside some for this and that. Also for the pamong [municipal police] for about 100 [thousand], "here's the grant", I went to the village office. "Please meet and greet with the pamongs first.""

Code: Getting/obtaining money from the project
REC11 checked_translated 171104

4.

"So from that 8 spots, maybe I only took 10 million. 800 [million] was taken by Mr. X. Then it was shared within the provincial government, to the treasury director, to the council, those were the ones who approved the proposal. "

Code: Getting/obtaining money from the project
REC11 checked_translated 171104

5.

" Tapi dengan adanya kasus ini itu saya kan kena juga. Kenanya itu terima fee 50 juta yang saya ceritakan tadi, itu sudah termasuk mendapatkan to? "

Code: Getting/obtaining money from the project
REC12

6.7.4. Taking money from the government budget

1.

"Where the money go, and I was only ordered to make a fictious report that the money has been spent on such and such. They asked the same thing in 2013, starting from that point... Well the signature was mine, why didn't I get any and I didn't feel that they returned the money and none of it was channeled to the government.)

P: He'em he'em he'em

(Uh huh.)

R: Lha mulai dari situ, saya ikut-ikutan akhirnya. Hehehehe."

Code: Taking money from the government budget
REC10_translated_checked

2.

"I knew that I was being used from that point and in 2013, I took it all. They did not know that. They only know the data. From people's tax, I gathered it, then I made the report, I input it. I had the transaction codes of the bank. They knew nothing about the money. First of all, honestly, I bought a house in a citry, sir. For my wife, I bought it for 800 million. Taken from that account, in 2013. "So this is how it works". They took the cash, while I took [manipulated] the data."

Code: Taking money from the government budget
REC10_translated_checked

3.

"So I just took it, like the Javanese says "Wes kadung teles nyemplung ae wes" [I got myself into it already, why don't I just go even further]. Right? You understand Javanese, right"

Code: Taking money from the government budget
REC10_translated_checked

4.

" They took another billion and more, I took the rest 800. So there wasn't any cash deposited from district x. I took the rest of it. I took all of it. I made a fool out of them this time"

Code: Taking money from the government budget
REC10_translated_checked

5.

"“Yes, sir! Just sign here, sir”. “Oh, okay”. Convinced for two times, they trusted on me. They took around 1 billion, I took 2 [billion] more. They traced my bank accounts, out of 6 accounts. There were none of them on my accounts. I took cash from it. So I know the process of how to withdraw it [the money]."

Code: Taking money from the government budget
REC10_translated_checked

6.7.5. Mark-up

1.

" Sana mintanya juga banyak, feenya itu. Mau ga mau harus dimark-up harga itu, harganya dua setengah dibuatlah empat. "

Code: Mark-up
REC06_translated_checked

2.

" Ya, APBD. Daerah ya. 25 diambil sana gimana pertanggungjawabkannya. Saya kan harus mempertanggungjawabkan 100 kan. Ketika diperiksa kok cuma 50, gimana? Lah memang 50 kenyataannya saya terima. Ya saya harus markup. "

Code: Mark-up
REC19

3.

"Yes, we came from the same district, hang out together. And if we for example wanted to mark-up some pricing, we share it.)
P: Ke?
(To?)
R: Ke satu kecamatan, seperti saya iya, ada untuk camat sendiri, kita ga ada dana, maksutnya tidak ada dana dalam anggaran dana kita. Tapi kita harus bisa ngasihkan ke camat, ngasihkan ke keteman kita, ketua, FK, semuanya. Itu kita harus, saya bendahara harus itu.
(To a certain district, just like me, for the district hear. Let’s say we don’t have the budget, but we need to allocate some [money] to our district head, to our friends, the chief, district facilitator, everyone. We should, I, the treasurer, should do it.)
P: Menaikan?
(Marking up?)
R: Misalnya photocopy 5000 jadi 10.000 dan itu pasti kita bagi, kita bagi.
(Let’s say the photocopy costs 5000, [we] changed it to 10.000, we definetely share it.)"

Code: Mark-up
REC32_translated_checked

4.

"Simpan pinjam. Kalau fisik kita tidak bisa mark-up dana kan. Kita ngasih dari kecamatan ke desa misalkan 10 juta, jadi aparatur juga harus 10 juta. Kita kalau mau me mark-up, kalau ada pelatihan-pelatihan itu bisa kita me-mark-up dana dari SPP, itu bisa. Jadi macetnya nya itu saya di SPPnya aja, kalau fisik ga bisa.
(Savings and loan. For the public facility, we were unable to mark-up the payments. From the district to village we gave in 10 million, so the apparatus should be 10 million as well. If we would like to mark it up, we were able to derive it from the trainings [budget]). So the bad transaction was in the savings and loan."

Code: Mark-up
REC32_translated_checked

6.7.6. Getting money from the failed credit

1.

Code: Getting money from the failed credit
REC31
