# Supplementary material for: Exploring the path to corruption–An informed grounded theory study on the decision-making process underlying corruption
Source: PLoS One. 2023 Sep 21;18(9):e0291819. doi: 10.1371/journal.pone.0291819 (PMC10513331; doi:10.1371/journal.pone.0291819)
Supplement: Attachment — Submitted filename: Response to Reviewers.docx [file pone.0291819.s004.docx]

**Response Letter**

Dear Prof. Baogui Xin,

Thank you for giving us the opportunity to address some minor revisions. We have adapted our manuscript based on your and the reviewers’ comments. Below are our responses to your and reviewers’ comments and suggestions.

**Responses to the Comments of the Editor**

**Journal requirements:**

*Please review your reference list to ensure that it is complete and correct. If you have cited papers that have been retracted, please include the rationale for doing so in the manuscript text, or remove these references and replace them with relevant current references. Any changes to the reference list should be mentioned in the rebuttal letter that accompanies your revised manuscript. If you need to cite a retracted article, indicate the article’s retracted status in the References list and also include a citation and full reference for the retraction notice.*

Response: Thank you for reminding us to check our reference list. We have checked our references once more and can conclude that the reference list is complete and correct. We also checked our references through Retraction Watch Database (<http://retractiondatabase.org>) and found no retracted articles in our reference list. While we have been thorough in our search, we appreciate your specific guidance if we would have unadvertedly cited a retracted manuscript.

**Responses to the Comments of Reviewer #1**

*General comment –I appreciate the effort you have put into revising and improving your manuscript titled "Exploring the path to corruption – An informed grounded theory study on the decision-making process underlying corruption." The revisions you have made in response to the previous round of review have indeed enhanced the overall quality of your work.
That said, upon careful consideration of this current version, I still see some areas that could use a bit more refinement. These are minor issues and mainly pertain to details that would further improve the clarity and completeness of your study. I am confident that you will be able to address these issues swiftly, as they do not fundamentally alter the scientific content of your work. I am looking forward to receiving your revised manuscript and to seeing this important piece of research ready for publication.*

Response: Thank you for your positive feedback regarding our revisions. Indeed, we found that your constructive feedback has helped us a lot to improve the quality of our manuscript. We would also like to thank you for providing some additional minor comments on this current version. Please find our responses to your current comments below.

*Comment 1 – I've reviewed the updated data availability statement as well as your responses to both me and the editor. I understand your concerns about participant confidentiality in the context of your research on corruption, which indeed deals with sensitive issues. This is an important consideration and certainly a challenge when dealing with such a delicate topic.
However, the principles of data availability are central to the progress of science, which include transparency, verification of results, fostering new research, economic responsibility, skill development, and public trust. These principles apply to all fields of research and play a crucial role in maintaining the credibility and reproducibility of scientific work.
While I fully understand your point of view regarding the interview transcripts, perhaps there is an alternative solution to consider. If providing anonymised transcripts of interviews is not feasible due to concerns over possible identification of participants, perhaps you could consider sharing your field notes, if these are available. They could be edited and anonymised to a greater extent than interview transcriptions and may provide sufficient data to allow replication of your findings without breaching confidentiality agreements with your participants.
I would like to emphasize that this suggestion is in line with maintaining the balance between respecting participant confidentiality and upholding the standards of data availability in scientific research.*

Response: Unfortunately, we did not create any specific “field notes” during our research (please note that this may depend on the qualitative approach taken). We would like to offer to share our coding dictionary in the Supporting Information, however, to adhere to the transparency standards of good research. As we indicated in the manuscript (lines 386-399, p. 17), the interviews were done in the Indonesian language and were coded in original transcripts and audio recordings. Therefore, most of the quotes in the coding dictionary are in the Indonesian language. Some of them were translated into English to let readers who do not speak Indonesian have some impressions about the coding process.

*Comment 2 – While I appreciate the authors' attempt to explain the rationale for including Indonesian informants in the study, I believe the language used in lines 305-308 on page 13 may inadvertently stigmatise a nation. The statement "Indonesia can be considered a corrupt country" is a potentially stigmatising label that may paint an unbalanced picture of the situation in Indonesia. I understand the authors’ intent to emphasize the prevalence of reported corruption cases in Indonesia as an explanation for their choice of sample; however, this framing could be perceived as negative or offensive.
Corruption is a global issue and exists in varying degrees in every country. Using an index score to label an entire country as "corrupt" oversimplifies a complex issue. It would be more accurate and sensitive to refer to Indonesia's corruption perception index (CPI) scores, indicating a high incidence of reported corruption cases, rather than branding the entire nation as "corrupt."
As an alternative, the authors could consider using a more neutral language, such as "Due to the consistently low Corruption Perception Index (CPI) scores and frequent media reports of corruption cases at various levels of government, Indonesia was selected as the focal context for this study." This rephrasing provides the necessary justification for the authors' sample choice while avoiding potentially harmful stereotyping.*

Response: Thank you for noticing that our language in lines 305-308 page 13 potentially stigmatizes the country and could be perceived as negative and offensive. We appreciate the suggested rephrasing sentences to avoid stigmatizing Indonesia as a corrupt country. We have now changed the sentences based on your suggestion (lines 305-307, page 13).

**Responses to the Comments of Reviewer #2**

*The authors have adequately addressed comments raised in a previous round of review. In the manuscript，authors explored new forms of corrupt decision-making elements within this process, and discovered some important cognitive processes that lead up to corrupt actions. Although the manuscript has some shortcomings in the innovation of its conclusions, it has some value in the methodology of studying corruption through grounded theory.*

Response: Thank you for your positive feedback. Your valuable comments have provided important insights for us to enhance our work.
